# A Systematic Review and Meta-analysis of the Effectiveness of Remdesivir to Treat SARS-CoV-2 in Hospitalized Patients: Have the Guidelines Evolved With the Evidence?

**DOI:** 10.1093/cid/ciaf111

**Published:** 2025-03-11

**Authors:** Michele Bartoletti, Essy Mozaffari, Alpesh N Amin, Yohei Doi, Paul Loubet, Christina G Rivera, Michael Roshon, Aaditya Rawal, Emily Kaiser, Maria Vutcovici Nicolae, Shuai Fu, Thomas F Oppelt, Mel Chiang, Paul E Sax, Andre C Kalil

**Affiliations:** Department of Biomedical Sciences, Humanitas University, Milan, Italy; Infectious Disease Unit, IRCCS Humanitas Research Hospital, Milan, Italy; Medical Affairs, Gilead Sciences, Foster City, California, USA; Division of Hospital Medicine & Palliative Medicine, University of California Irvine, Irvine, California, USA; Departments of Microbiology and Infectious Diseases, Fujita Health University School of Medicine, Toyoake, Aichi, Japan; Division of Infectious Diseases, University of Pittsburgh School of Medicine, Pittsburgh, Pennsylvania, USA; Department of Infectious Diseases, Centre Hospitalier Universitaire de Nîmes, Nîmes, France; Department of Pharmacy, Mayo Clinic, Rochester, Minnesota, USA; Department of Quality, UC Health-Southern Region, Colorado Springs, Colorado, USA; US Market Access and Health Economics, Costello Medical, Boston, Massachusetts, USA; US Literature Reviews, Costello Medical, Boston, Massachusetts, USA; Certara Drug Development Solutions, Certara, New York, New York, USA; Certara Drug Development Solutions, Certara, New York, New York, USA; Medical Affairs, Gilead Sciences, Foster City, California, USA; Medical Affairs, Gilead Sciences, Foster City, California, USA; Division of Infectious Diseases, Brigham and Women’s Hospital and Harvard Medical School, Boston, Massachusetts, USA; Division of Infectious Diseases, University of Nebraska Medical Center, Omaha, Nebraska, USA

**Keywords:** remdesivir, SARS-CoV-2, treatment guidelines, effectiveness, COVID-19

## Abstract

**Background:**

With progressive accumulation of knowledge on SARS-CoV-2 infection clinical management, treatment guidelines recommended several options including remdesivir, a broad-spectrum antiviral. Given the evolving nature of coronavirus disease 2019, capturing the totality of scientific evidence from clinical trials and observational studies is critical to inform clinical decision making. We conducted a systematic literature review with meta-analysis to summarize the effectiveness of remdesivir among hospitalized adults.

**Methods:**

We systematically searched MEDLINE, Embase and Cochrane Library databases for interventional and observational studies examining remdesivir efficacy. A rigorous double-reviewer approach was used for source identification, screening, data extraction and risk of bias assessment. A hierarchical random-effects model meta-analysis was used, with subgroup analyses for randomized controlled trials (RCTs) and real-world (RW) studies.

**Results:**

From January 2019 to December 2023 >18 000 sources were screened, and 122 unique studies were identified, reporting on 25 174 participants in RCTs and 1 279 859 in RW studies. Remdesivir significantly increased survival in the overall population (odds ratio, 0.69 [95% confidence interval, .55–.86]; *P* = .001] across SARS-CoV-2 variants and disease severity levels: no supplemental oxygen (0.81 [.75–.88]), low-flow oxygen (0.71 [.64–.79]), high-flow oxygen (0.87 [.83–.91]), and invasive mechanical ventilation (0.78 [.68–.90]). Rehospitalization risk was significantly reduced in patients receiving remdesivir (odds ratio, 0.72 [95% confidence interval, .64–.81]).

**Conclusions:**

Our comprehensive systematic literature review, capturing the totality of evidence, showed a significant survival benefit among patients hospitalized for SARS-CoV-2 infection and receiving remdesivir, across all disease severity levels. To assure that healthcare providers are aware of and deploy evidence-based optimal care, recommendations should rely on both RCT and RW data.

## Teaser

SARS-CoV-2 infection treatment guideline recommendations have not been updated in parallel with evolving evidence. Remdesivir is associated with higher survival rates and reduced rehospitalization risk in both randomized controlled trials and real-world studies. Updating the guidelines with the totality of evidence is essential to improve survival outcomes.

## Graphical Abstract

**Graphical Abstract:**
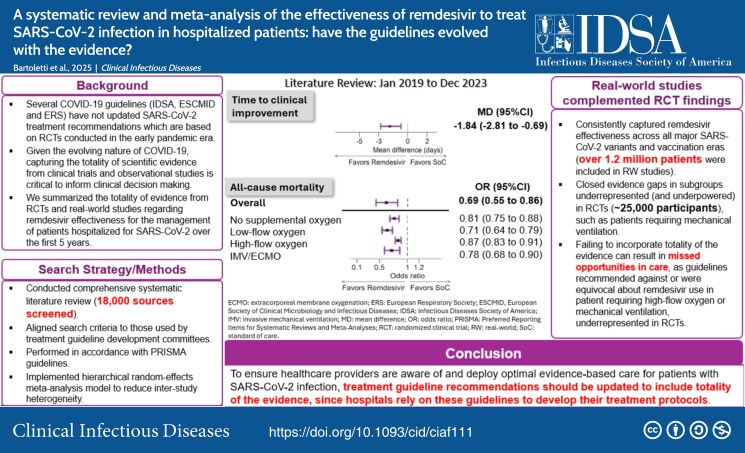
This graphical abstract is also available at Tidbit: https://tidbitapp.io/tidbits/a-systematic-review-and-meta-analysis-of-the-effectiveness-of-remdesivir-to-treat-sars-cov-2-infection-in-hospitalized-patients-have-the-guidelines-evolved-with-the-evidence-b65f4479-6953-470d-a614-d654cefd6136?utm_campaign=tidbitlinkshare&utm_source=ITP

Clinical practice guidelines (CPGs) aim to reduce variability in practice in order to improve the quality of care by establishing best practice standards [1, 2]. With no definitive standardized approach to developing CPGs, there are inconsistencies in identifying, appraising, and synthesizing the clinical evidence [1, 3, 4]. While randomized clinical trials (RCTs) are best suited to demonstrate efficacy of an intervention under controlled settings [5], their stringent inclusion/exclusion criteria may prevent the generalizability of findings to more heterogeneous real-world (RW) patient populations [6, 7]. An assessment of clinical trial representativeness to RW populations was recently performed using the Clinical Practice Research Datalink GOLD database in England [7]. Data on 989 unique drugs prescribed for 286 conditions from 43 895 clinical trials among 5 685 738 individuals indicated that population subgroups such as adolescents and elderly persons are often excluded from clinical trials, despite making up about 50% of the population [7]. Similarly, patients with comorbid conditions or receiving concomitant medication are frequently excluded from trials despite making up 67.7% and 98.5% of the population, respectively [7]. As it would be unfeasible to conduct RCTs in all subgroups of interest and unethical to restrict access to approved and safe medications, RW studies performed after RCTs completion and regulatory approval can be leveraged to fill the gaps in evidence-based medicine [6–8].

The coronavirus disease 2019 (COVID-19) pandemic presented a new challenge to healthcare systems worldwide. The rapid pace of the pandemic created a pressing need for guidance in clinical decision making in an era when scientific evidence was lacking [9]. Evidence from RCTs emerged in 2020, months after the pandemic started, and clinical management guidelines were developed based on these early research findings.

Remdesivir is a broad-spectrum antiviral, approved by the United States and European authorities in 2020 as the first anti–COVID-19 treatment for hospitalized patients [10, 11]. Treatment with remdesivir for mild to severe COVID-19 in immunocompetent patients not requiring invasive mechanical ventilation is recommended by the most prominent international, North American, and European CPGs, based on RCT findings (Supplementary Table 1) [12–17]. Adolescents, pregnant or breastfeeding women, and people with liver or renal impairment are population groups excluded from RCTs assessing remdesivir effectiveness in hospitalized patients. There has been a progressive growth in understanding the natural history of severe acute respiratory syndrome coronavirus 2 (SARS-CoV-2) infection, and RW studies contributed to evidence for management strategies across the range of patient segments, severity levels, and pandemic to endemic eras.

The current review aims to ascertain how the scientific evidence for the effectiveness of remdesivir in SARS-CoV-2 infection management of hospitalized patients has evolved through the pandemic and endemic eras by summarizing the totality of evidence from RCTs and RW studies through a comprehensive systematic literature review (SLR).

## METHODS

### Search Strategy

We conducted an iterative systematic review of the literature published from January 2019 to December 2023 to identify randomized (ie, interventional) and observational (ie, noninterventional) studies reporting remdesivir efficacy in hospitalized patients with SARS-CoV-2 infection. Our review was performed in accordance with the Preferred Reporting Items for Systematic Reviews and Meta-Analyses (PRISMA) guidelines [18]. MEDLINE, including MEDLINE In-Process, MEDLINE In-Data-Review, and MEDLINE Epub Ahead of Print, and Embase databases were searched via the Ovid SP platform in December 2023 (Supplementary Tables 2 and 3). The Cochrane Database of Systematic Reviews and the Cochrane Central Register of Controlled Trials (CENTRAL) were searched via Wiley Online platform. Gray literature reviews were performed in January 2024 (Supplementary Table 4). Bibliographies of relevant SLRs were hand searched for relevant studies.

### Inclusion Criteria

Observational and interventional studies reporting efficacy of remdesivir in hospitalized patients with SARS-CoV-2 infection were eligible for inclusion (Supplementary Table 5). The search results were manually deduplicated. There are variations in defining observational studies across countries [19]. For simplicity we used the terms *RCTs* for interventional studies and randomized clinical trials, and *RW studies* for noninterventional, observational studies.

### Literature Screening, and Data Extraction

A dual-reviewer approach was used for abstract screening, full-text review, and data extraction (Supplementary Figure 1). Discrepancies were discussed between reviewers until consensus was reached. Study and patient characteristics, treatments, and efficacy outcomes were extracted.

### Risk of Bias Assessment

Quality was appraised for RCTs using the University of York Centre for Reviews and Dissemination (CRD) guidelines [20] for interventional studies (Supplementary Table 6) and for RW studies using the Downs and Black checklist [21] for noninterventional studies (Supplementary Table 7). The checklist assesses quality of reporting (9 items), external validity (3 items), internal validity (bias and confounding [7 items]), and power (1 item). Risk of bias assessment was completed by one reviewer and verified by a second.

### End Points

We assessed key end points used by CPGs to appraise the evidence for remdesivir treatment recommendations in hospitalized adults with SARS-CoV-2 infection: all-cause mortality (defined as the number of inpatient deaths at 28–30 days), time to clinical improvement (the number of days between admission and the day clinical improvement criteria were fulfilled), time to clinical recovery (the number of days between admission and the day clinical recovery criteria were fulfilled), and rehospitalization (the number of patients requiring rehospitalization after hospitalization for SARS-CoV-2 infection).

### Statistical Analysis

Due to heterogeneity in reporting outcomes we grouped studies into categories with similar characteristics. Mild-moderate disease was grouped with those not requiring oxygen support, severe disease with those requiring noninvasive ventilation if use of low-flow oxygen (LFO) or high-flow oxygen (HFO) was reported, and critical disease was grouped with those requiring invasive mechanical ventilation. Studies reporting mortality at 28, 29 or 30 days were grouped together.

A feasibility assessment was conducted to assess suitability of the identified studies for meta-analysis. We performed a statistical heterogeneity test by calculating *I*^2^ [22], Cochran's heterogeneity *Q* statistic, and its *P* value [23] for pairwise comparison remdesivir versus no remdesivir/placebo/standard of care for each end point.

A hierarchical random-effects meta-analysis was used to generate pooled summaries of remdesivir efficacy and effectiveness for each end point. We evaluated the effect of remdesivir compared with no remdesivir/placebo/standard of care by differentiating variability within RW studies and RCTs (see Supplementary Methods). Studies not comparing the impact of treatment between remdesivir and no-remdesivir groups for overall population or by oxygen support were excluded from analyses. The numbers of events in treated and untreated groups were used to compute odds ratios (ORs) for binary end points or mean difference for continuous end points.

First, we conducted a meta-analysis for the overall population (ie, not stratified by oxygen support requirement. Next, the weighted average across oxygen support (no supplemental oxygen, LFO, HFO, and invasive mechanical ventilation/extracorporeal membrane oxygenation) was incorporated. Pooled results were adjusted for duration of remdesivir treatment and oxygen support at baseline. All analyses accounted for RCT and RW studies separately. The nlme R package (version 4.2.3) was used for statistical analyses.

## RESULTS

**Figure 1.**
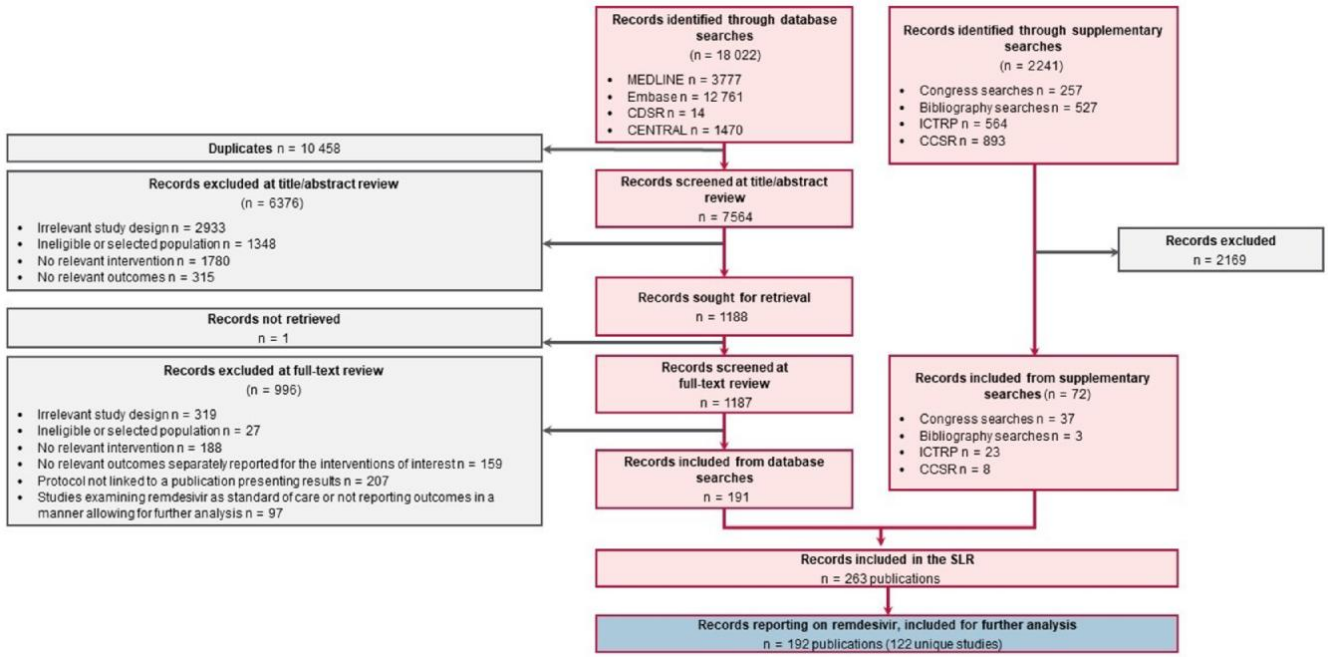
PRISMA (Preferred Reporting Items for Systematic Reviews and Meta-Analyses) flow diagram. Any article that did not examine remdesivir as a therapy of interest was excluded from analysis. Abbreviations: CCSR, Cochrane COVID-19 Study Record; CDSR, Cochrane Database of Systematic Reviews; CENTRAL, Cochrane Central Register of Controlled Trials; COVID-19, coronavirus disease 2019; ICTRP, International Clinical Trials Registry Program; SLR, systematic literature review.

The systematic literature search resulted in 18 022 relevant sources, including 3777 in MEDLINE, 12 761 in Embase, 14 in the Cochrane Database of Systematic Reviews, and 1470 in CENTRAL. In addition, 2241 sources were identified through supplementary searches of congress proceedings, the World Health Organization (WHO) International Clinical Trials Registry Platform, the Cochrane COVID-19 Study Record, and the bibliography of identified SLRs. A total of 192 publications were retained, including peer-reviewed articles, conference abstracts and posters stemming from 122 unique studies (21 RCTs and 101 RW studies) (Figure 1).

Included studies were conducted between February 2020 and April 2022 (complete list provided in Supplementary Table 15). There were 25 174 participants enrolled in RCTs and 1 279 859 in RW studies. Most RCTs (61.9%) enrolled participants from multiple countries; a minority of RW studies (3%) spanned multiple countries. Both RCT and RW studies covered similar geographic regions (Supplementary Figure 2).

### End Points

#### Mortality

Of the 122 unique studies included, 108 reported number of deaths, mortality rate, or risk of mortality. Only 21 studies (4 RCTs and 16 RW studies) comparatively assessed the mortality risk at 28–30 days between remdesivir and no-remdesivir groups overall or by oxygen support at admission (characteristics summarized in Table 1). While some RCTs' subgroups and RW studies with small sample size showed inconclusive results regarding the impact of remdesivir on mortality, appropriately powered RW studies showed a significant survival benefit in remdesivir-treated patients across disease severity levels and COVID-19 variant periods (Supplementary Table 8).

**Table 1.** Characteristics of Studies Reporting Remdesivir Efficacy and Effectiveness on Mortality in Hospitalized Adults With Coronavirus Disease 2019

Authors (Year) [Reference]	Study Period	Location and Setting	Study Design	Sample Size, No. of Patients	Patients Not Requiring Supplemental Oxygen at Baseline,%	Duration of Remdesivir Treatment, Days	Primary Efficacy Outcome
**Bechman et al (2022) [24]**	Mar 2020–Feb 2021	UK; multicentric	RW study, prospective	3949	NR	NR	Mortality rate at 28 d
**Beigel et al (2020) [25] (ACTT-1 study)**	Feb–May 2020	Denmark, Germany; Greece, Japan, Korea, Mexico, Singapore, Spain, UK, and US; multicentric	RCT	1062	12 (Treated), 14 (Untreated)	Up to 10^a^	Time to recovery
**Benfield et al (2021) [26]^b^**	Feb–Dec 2020	Denmark; multicentric	RW study, retrospective	2747	NR	NR	Survival status at 30 d; mechanical ventilation
**Breskin et al (2023) [27]^c^**	May 2020–Dec 2021	US; multicentric	RW study, retrospective	71 068	NR	NR	Mortality rate at 30 d; incidence of IMV/ECMO
**Caffrey et al (2023) [28]^c^**	May 2020–Nov 2021	US; multicentric	RW study, retrospective	18 874	17.7 (Treated), 15.9 (Untreated)	NR	Time to inpatient death
**Chokkalingam et al (2022) [29]^c^**	May 2020–May 2021	US; multicentric	RW study, retrospective	113 579	64.2	NR	Time to inpatient death
**De Vito et al (2022) [30]^c^**	Aug 2020–Oct 2021	Italy; multicentric	RW study, retrospective	1080	NR	NR	Mortality rate at 28 d
**Diaz et al (2022) [31]^c^**	Feb–May 2020	US; multicentric	RW study, retrospective	1138	37.4 (Treated), 36.3 (Untreated)	5 or 10	Overall survival
**Dobrowolska et al (2023) [32]^c^**	Aug 2021–Apr 2022	Poland; multicentric	RW study, retrospective	1822	NR	5 or 10	Need for oxygen therapy; need for mechanical ventilation; mortality rate at 28 d
**Finn et al (2022)** **[33]**	Apr–Dec 2020	US; multicentric	RW study, prospective	2230	NR	NR	LOS; 30-d readmission; postdischarge 30-d mortality rate
**Garibaldi et al (2022) [34]^c^**	Feb 2020–Feb 2021	US; multicentric	RW study, retrospective	36 656	15.6 (Treated) 13.5 (Untreated)	5	Time to clinical improvement
**Grundmann et al (2023) [35]**	Jan 2020–Jun 2021	UK; multicentric	RW study, prospective	89 297	NR	NR	Risk of neurological complications
**Lapadula et al (2020) [36]**	Mar 2020–Mar 2020	Italy; single center	RW study, retrospective	113	0	10	Time to death; time to hospital discharge
**Leding et al (2023) [37]^b^**	Feb 2020–Apr 2021	Denmark; multicentric	RW study, retrospective	3826	56.2 (Treated), 55.4 (Untreated)	NR	Use of IMV; mortality rate at 30 d
**Marx et al (2023) [38]**	Jul 2020–Jun 2021	Germany; single center	RW study, retrospective	839	13.6 (Treated), 11.4 (Untreated)	NR	Time to clinical improvement
**Mozaffari et al (2022) [39]^c^**	Aug–Nov 2020	US; multicentric	RW study, retrospective	45 542	27.6 (Treated) 27.6 (Untreated)	NR	Inpatient mortality rate at 14 and 28 d
**Mozaffari et al (2023) [40]**	Dec 2020–Apr 2022	US; multicentric	RW study, retrospective	213 264	35 Overall	NR	Mortality rate at 14 and 28 d
**Olender et al (2021) [41] (SIMPLE-Severe study)**	Feb–May 2020	US, Italy, Spain, Germany, Hong Kong, Singapore, South Korea, and Taiwan; multicentric	RCT	1767	14.1 (Treated) 14.1 (Untreated)	5 or 10^d^	Clinical recovery at 14 d; mortality rate at 28 d
**Henao-Restrepo et al (2022) [42] (Solidarity study)**	Mar 2020–Jan 2021	Albania, Austria, Belgium, Finland, France, Ireland, Italy, Lithuania, Luxembourg, North Macedonia, Norway, Spain, Switzerland, Argentina, Brazil, Colombia, Honduras, Peru, Egypt, India, Indonesia, Iran, Kuwait, Lebanon, Malaysia, Pakistan, Philippines, Saudi Arabia, and South Africa; multicentric	RCT	14 304	NR	10	Mortality rate at 28 d
**Wang et al (2020) [43]^c^**	Feb–Mar 2020	China; multicentric	RCT	236	0 (Treated) 4 (Untreated)	10	Time to clinical improvement

Abbreviations: ECMO, extracorporeal membrane oxygenation; IMV, invasive mechanical ventilation; LOS, length of hospital stay; NR, not reported; RCT, randomized controlled trial; RW, real-world; UK, United Kingdom; US, United States.

^a^Patients were randomized to either remdesivir (200 mg loading dose on day 1, followed by 100 mg/d for up to 9 additional days) or placebo for up to 10 days [25].

^b^Corticosteroids were used in all patients receiving remdesivir.

^c^Corticosteroids were used in both remdesivir and no-remdesivir groups.

^d^Patients were randomized to remdesivir 200 mg on day 1 followed by remdesivir 100 mg/d either on days 2–5 or on days 2–10 [41].

**Figure 2.**
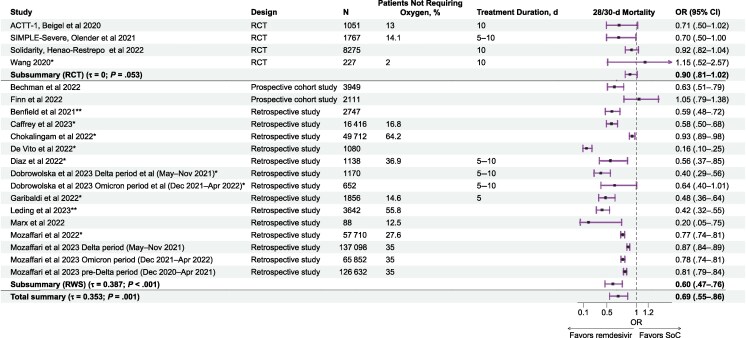
Forest plot for mortality at 28–30 days in hospitalized patients with coronavirus disease 2019, overall population [24–26, 28–34, 37–43]. *Corticosteroids were used in both remdesivir and no-remdesivir groups. **Corticosteroids were used in all patients receiving remdesivir. Note that the odds ratios (ORs) reported in the plot resulted from the meta-analysis based on the number of events reported in the original studies. The Solidarity trial assessed in-hospital mortality regardless of whether deaths occurred before or after day 28. Abbreviations: CI, confidence interval; RCT, randomized controlled trial; RWS, real-world study; SoC, standard of care.

The pooled results of the meta-analysis of the 17 publications included in the overall population analysis (4 RCTs and 13 RW studies) indicated a significant survival benefit among patients receiving remdesivir treatment (OR, 0.69 [95% confidence interval (CI), .55–.86]; *P* = .001) (Figure 2). The subgroup analyses on survival benefit showed ORs of 0.60 (95% CI, .47–.76; *P* < .001) for RW studies and 0.90 (.81–1.02; *P* = .053) for RCTs. The DisCoVeRy trial, an add-on to the WHO Solidarity trial, was not included in the final analysis to avoid double-counting participants, as test analyses showed no change of the main results when DisCoVeRy was added.

**Figure 3.**
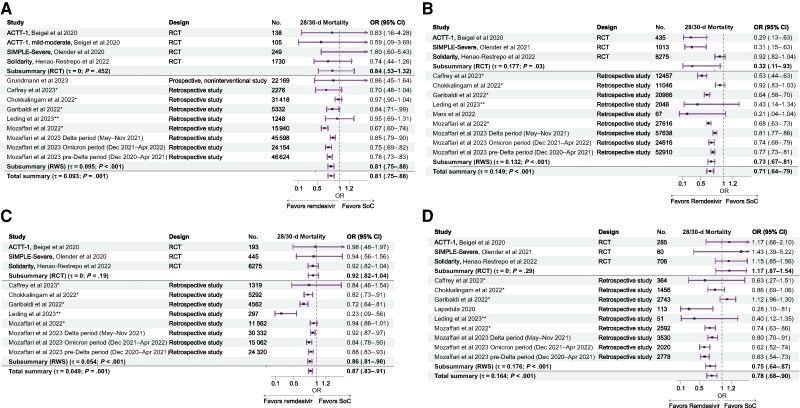
Forest plot for mortality at 28–30 days in hospitalized patients with coronavirus disease 2019, by oxygen support requirement at admission [25, 28, 29, 34–42]. Results are grouped by oxygen requirement at admission: no supplemental oxygen (*A*), low-flow oxygen (*B*), high-flow oxygen (*C*), or invasive mechanical ventilation/extracorporeal membrane oxygenation (*D*). *Corticosteroids were used in both remdesivir and no-remdesivir groups. **Corticosteroids were used in all patients receiving remdesivir. Note that the Solidarity trial was not designed to differentiate the impact of remdesivir treatment between patients receiving low-flow or high-flow oxygen support at admission. In-hospital mortality was assessed regardless of whether deaths occurred before or after day 28. The odds ratio (ORs) reported in the plots resulted from the meta-analysis based on the number of events reported in the original studies. Abbreviations: CI, confidence interval; RCT, randomized controlled trial; RWS, real-world study; SoC, standard of care.

The analyses stratified by oxygen support at admission indicated a significant survival benefit across all levels, for no supplemental oxygen (OR, 0.81 [95% CI, .75–.88]), LFO (0.71 [.64–.79]), HFO (0.87 [.83–.91]), and invasive mechanical ventilation/extracorporeal membrane oxygenation (0.78 [.68–.90]) (Figure 3). The subgroup analyses indicated that the survival benefit was significant across all levels of oxygen support in RW studies, while RCTs showed a significant impact on survival among patients receiving LFO (OR, 0.32 [95% CI, .11–.93]).

#### Time to Clinical Improvement

Clinical improvement was reported in 10 unique studies, of which 6 (3 RCTs and 3 RW studies) comparatively assessed time to clinical improvement between remdesivir and no-remdesivir groups (Supplementary Tables 9 and 10). There was substantial heterogeneity in defining clinical improvement, each of the included studies using different criteria. The meta-analysis results for the overall population indicated a significant impact of remdesivir on time to clinical improvement overall (mean difference, −1.84 [95% CI, −2.81 to −.69]) as well as in analyses stratified by RCTs (−2.98 [−3.01 to −1.09]) and RW studies (−2.20 [−3.43 to −.67]) (Supplementary Figure 3).

#### Time to Clinical Recovery

Clinical recovery was reported in 13 unique studies, of which 3 (1 RCT and 2 RW studies) comparatively assessed the time to clinical recovery between remdesivir and no-remdesivir groups (Supplementary Tables 11 and 12). The meta-analysis results for the overall population indicated a nonsignificant impact of remdesivir on the time to clinical recovery (mean difference, −0.31 [95% CI, −3.37 to 3.94]).

#### Rehospitalization

Of 9 unique studies reporting rehospitalization, only 4 (1 RCT and 3 RW studies) comparatively assessed the 30-day risk of rehospitalization between remdesivir treated and untreated groups (Supplementary Tables 13 and 14). The meta-analysis results for the overall population indicated a significant reduction in risk of rehospitalization in patients receiving remdesivir (OR, 0.72 [95% CI, .64–.81]), mostly driven by the results reported in RW studies (0.70 [.63–.78]) as the RCT results were inconclusive for this outcome (Supplementary Figure 4).

### Heterogeneity and Risk of Bias

Of the 4 RCTs evaluated in the overall population mortality analysis, the ACTT-1 study [25] had the lowest risk of bias in all categories except 1, while the other 3 RCTs had ≥1 factor contributing to a higher risk of bias in results. The 13 RW studies were of moderate to low risk of bias, and each had ≥1 subelement that could not be evaluated for bias due to insufficient information, with 12 of 13 RW studies not reporting details on statistical power calculations. However, all included studies were appropriately validated and evaluated hospitalized patients with diagnosed SARS-CoV-2 infection, confirming that the studies had appropriate populations for analysis.

## DISCUSSION

Our comprehensive SLR was designed to capture the totality of evidence in a guideline-grade robust approach. More than 18 000 sources were reviewed, and 122 unique studies reporting remdesivir effectiveness enrolling 25 174 participants in RCTs and 1 279 859 in RW studies were included. Evidence accumulated during the pandemic covered the full range of disease severity and continued to grow in the postpandemic era. Remdesivir had a significant impact on key clinical outcomes commonly used to summarize the evidence for the development of CPG recommendations [12, 13, 15, 16]. The totality of evidence from RCTs and RW studies indicates a significant survival benefit in patients hospitalized for SARS-CoV-2 infection and treated with remdesivir, both overall and across disease severity levels, as assessed by oxygen support requirement at admission, a significantly lower risk of hospital readmissions and shorter time to clinical improvement. Analyses stratified by study design indicated that the effectiveness of remdesivir on key clinical outcomes was consistently captured by RW studies. CPG recommendations for remdesivir use are based almost exclusively on results from RCTs conducted at the beginning of the pandemic, which indicate a significant survival benefit of remdesivir, mainly for the subgroup of hospitalized patients not requiring invasive ventilation, which were the majority of patients, while the small subgroup of ventilated patients remained underpowered to detect survival outcomes. The Infectious Diseases Society of America, European Society of Clinical Microbiology and Infectious Diseases, and European Society Respiratory guidelines have not been updated since 2022 [12–15]. The WHO guidelines were updated fairly recently (November 2023) but still relied on the RCTs conducted in the pre-Omicron period for remdesivir recommendations [17]. Similarly, the 2024 National Institutes of Health recommendations for immunocompetent patients with SARS-CoV-2 infection were based exclusively on RCT efficacy results; RW effectiveness was considered only for recommendations applicable to patients with immunocompromising conditions, for whom the RCT data provided little insight [16].

Our SLR indicates that remdesivir RW effectiveness data became available in 2021, covering similar geographic regions as RCTs. As evidence continued to accumulate through late 2023, RW studies offered insights covering all major SARS-CoV-2 variants and vaccination eras, which could close the evidence gaps in certain population subgroups underrepresented (and underpowered for significance testing) in RCTs, such as critically ill patients requiring mechanical ventilation.

While RCTs constitute the highest-level evidence around efficacy [44] and are the preferred source to guide treatment recommendations, the use of large well-designed observational studies to provide important insights regarding effectiveness is relevant for both confirmation of RCT findings and generalizability to clinical practice. Indeed, observational studies were part of the evidence for recommendations regarding other SARS-CoV-2 infection treatments [15]. Specifically, the 2022 Infectious Diseases Society of America recommendations for the use of nirmatrelvir-ritonavir (updated March 2022), or convalescent plasma (updated February 2023) included findings from observational studies, but remdesivir recommendations (updated February 2022) did not [15]. The pandemic put considerable pressure on the development of guidelines to inform clinical practice and resulted in a high number of published CPGs. The systematic review by Burns et al [45] captured no less than 32 CPGs for the treatment of patients hospitalized for SARS-CoV-2 infections published in the first year of the pandemic, of which only 5 (15.6%) reported the search or screening strategy, performed evidence synthesis, or used a trained methodologist.

A 2024 comparison of WHO guidelines to national guidelines from 109 countries indicated significant global variations in treatment recommendations for SARS-CoV-2 infection [46]. Discrepancies in classification of disease severity, lack of guidance at the beginning of the pandemic, changes in WHO recommendations from one update to the next, inconsistencies in WHO appraisal of evidence, and lack of head-to-head comparison of available therapeutics were cited as potential reasons for the variations [46].

Four years after the start of the pandemic and 1 year after WHO declared that COVID-19 no longer constituted a global health emergency, there are new additional factors to consider for clinical decision making, such as the changing epidemiology potentially affected by changes in immunity due to vaccination(s), previous infection, or combination of the 2, and the associated shift in care from infectious disease to other medical specialties. The age of patients hospitalized for SARS-CoV-2 infection has significantly increased from the Alpha- and Delta-variant era to the Omicron era, and patients are now more likely to have comorbid conditions [47–49]. Racial and ethnic disparities in treatment were reported in several large RW studies in the United States [50–53]. As highlighted by the guidelines, the population subgroups at high risk of severe outcomes are those who are immunocompromised or elderly, those with comorbid conditions and polypharmacy, pregnant women, smokers, and those not vaccinated or not up to date with their vaccination [13, 15–17]. Yet these groups are usually not represented in clinical trials [7], but evidence to support guideline development could be gleaned from RW studies.

There are methodological difficulties in synthesizing evidence when studies with different designs, such as RCTs and observational studies, are included. A meta-epidemiological study of 102 meta-analyses published in journals with the highest impact factor indicated that 59% used random effects models and only 15% conducted subgroup analyses by study design; despite the substantial heterogeneity introduced by observational studies, no difference in effect estimates was found [54]. The pooled estimates in our sensitivity analysis for RW studies indicated a significant survival benefit in remdesivir-treated patients across all disease severity levels as defined by oxygen support, including invasive mechanical ventilation. Failing to incorporate evidence from RW studies likely resulted in missed opportunities in optimizing therapy for patients with critical SARS-CoV-2 infections requiring HFO or mechanical ventilation, as guidelines recommended against or were equivocal about the use of remdesivir in these patient categories. To assist clinicians in the decision making process, clinical guidelines should be translated to policies and hospital protocols.

The current study is an endeavor to summarize the totality of evidence for remdesivir efficacy and effectiveness for the treatment of SARS-CoV-2 infection among hospitalized patients. A hierarchical random-effects meta-analysis model was used, and sensitivity analyses were conducted separately for RCTs and RW studies to reduce interstudy heterogeneity. We assessed key end points used by CPGs to appraise the evidence for recommendations regarding remdesivir use in hospitalized adults with SARS-CoV-2 infection. The search criteria were aligned with the search strategies used by guideline development committees. Due to the low number of studies, it was not possible to assess remdesivir’s impact on other efficacy outcomes and by subgroup of disease severity. The analyses were adjusted for key effect modifiers, such as the proportion of population requiring oxygen support and the duration of treatment, but unreported confounders or inconsistencies in reporting confounders may have influenced the results. For example, study start year, publication time, and regions were not considered due to limited data availability.

In conclusion, to assure that healthcare providers are aware of and deploy evidence-based optimal care for patients with SARS-CoV-2 infection, international CPG recommendations should be updated to include all evidence, since many hospital protocol authors rely on guidelines for decisions at the local level. All efforts should be made to standardize the reporting of key clinical outcomes to facilitate assessment of the totality of evidence in developing and updating treatment guideline recommendations. Future research should expand this approach to all SARS-CoV-2 infection treatments and population subgroups of interest. Moreover, having updated guidelines can help future research assess compliance, efficacy, and outcomes with guideline-directed medical management.

## Supplementary Material

ciaf111_Supplementary_Data

## Supplementary Data

Supplementary materials are available at *Clinical Infectious Diseases* online. Consisting of data provided by the authors to benefit the reader, the posted materials are not copyedited and are the sole responsibility of the authors, so questions or comments should be addressed to the corresponding author.

## Notes

***Author contributions*.** E. M. and A. C. K. conceived the study. A. R. and E. K. curated the data and had access to the raw data. S. F. performed the formal analysis. M. B., E. M., A. N. A., Y. D., P. L., C. G. R., M. R., T. F. O., M. C., P. E. S. and A. C. K. supervised the study. M. V. N. and E.M. wrote the original draft of the manuscript. All authors reviewed and edited the manuscript. M. V. N. and S. F. validated the study data. A. C. K. had full access to all of the data and final responsibility for the decision to submit for publication.

***Acknowledgments.*** The authors thank Olena Polyakova for assistance with quality control.

***Data availability.*** Data are not publicly available.

***Financial support.*** This work was supported by Gilead Sciences.

## References

[1] De Leo A, Bloxsome D, Bayes S. Approaches to clinical guideline development in healthcare: a scoping review and document analysis. BMC Health Serv Res 2023; 23:37. 36647085 10.1186/s12913-022-08975-3 PMC9841716

[2] Zhang Y, Coello PA, Brożek J, et al Using patient values and preferences to inform the importance of health outcomes in practice guideline development following the GRADE approach. Health Qual Life Outcomes 2017; 15:52. 28460638 10.1186/s12955-017-0621-0 PMC5412036

[3] Benavidez G, Frakt AB. 2019. Fixing clinical practice guidelines. Health Affairs Forefront. Available at: https://www.healthaffairs.org/do/10.1377/forefront.20190730.874541/full/. Accessed 16 December 2024.

[4] Grol R, Cluzeau F, Burgers J. Clinical practice guidelines: towards better quality guidelines and increased international collaboration. Br J Cancer 2003; 89(suppl 1):S4–8. 12915896 10.1038/sj.bjc.6601077 PMC2753001

[5] Kalil AC . Treating COVID-19-off-label drug use, compassionate use, and randomized clinical trials during pandemics. JAMA 2020; 323:1897–8. 32208486 10.1001/jama.2020.4742

[6] Chew SY, Koh MS, Loo CM, Thumboo J, Shantakumar S, Matchar DB. Making clinical practice guidelines pragmatic: how big data and real world evidence can close the gap. Ann Acad Med Singap 2018; 47:523–7. 30636269

[7] Tan YY, Papez V, Chang WH, Mueller SH, Denaxas S, Lai AG. Comparing clinical trial population representativeness to real-world populations: an external validity analysis encompassing 43 895 trials and 5 685 738 individuals across 989 unique drugs and 286 conditions in England. Lancet Healthy Longev 2022; 3:e674–89. 36150402 10.1016/S2666-7568(22)00186-6

[8] Bierer BE, White SA, Barnes JM, Gelinas L. Ethical challenges in clinical research during the COVID-19 pandemic. J Bioeth Inq 2020; 17:717–22. 33169251 10.1007/s11673-020-10045-4 PMC7651825

[9] Lee CK, Merriam LT, Pearson JC, Lipnick MS, McKleroy W, Kim EY. Treating COVID-19: evolving approaches to evidence in a pandemic. Cell Rep Med 2022; 3:100533. 35474746 10.1016/j.xcrm.2022.100533 PMC8826498

[10] European Medicines Agency . 2020. Available at: https://www.ema.europa.eu/en/news/first-covid-19-treatment-recommended-eu-authorisation. Accessed 16 December 2024.

[11] Food and Drug Administration (FDA) . 2020. Available at: https://www.fda.gov/news-events/press-announcements/fda-approves-first-treatment-covid-19. Accessed 17 December 2024.

[12] Bartoletti M, Azap O, Barac A, et al European Society of Clinical Microbiology and Infectious Diseases guidelines for coronavirus disease 2019: an update on treatment of patients with mild/moderate disease. Clin Microbiol Infect 2022; 28:1578–90. 36028088 10.1016/j.cmi.2022.08.013 PMC9398787

[13] Bartoletti M, Azap O, Barac A, et al ESCMID COVID-19 living guidelines: drug treatment and clinical management. Clin Microbiol Infect 2022; 28:222–38. 34823008 10.1016/j.cmi.2021.11.007 PMC8606314

[14] Chalmers JD, Crichton ML, Goeminne PC, et al Management of hospitalized adults with coronavirus disease 2019 (COVID-19): a European Respiratory Society living guideline. Eur Respir J 2021; 57:2100048. 33692120 10.1183/13993003.00048-2021 PMC7947358

[15] Infectious Diseases Society of America . 2023. Available at: https://www.idsociety.org/practice-guideline/covid-19-guideline-treatment-and-management/. Accessed 12 December 2024.

[16] National Institutes of Health . 2024. Available at: https://iris.who.int/bitstream/handle/10665/365584/WHO-2019-nCoV-therapeutics-2023.1-eng.pdf?sequence=1. Accessed 16 December 2024.

[17] World Health Organization . 2023. Available at: https://iris.who.int/bitstream/handle/10665/365584/WHO-2019-nCoV-therapeutics-2023.1-eng.pdf?sequence=1. Accessed 12 December 2024.

[18] Page MJ, McKenzie JE, Bossuyt PM, et al The PRISMA 2020 statement: an updated guideline for reporting systematic reviews. BMJ 2021; 372:n71. 33782057 10.1136/bmj.n71 PMC8005924

[19] Leavy MB . Variations in how observational studies are defined. In: Multinational registries: challenges and opportunities. Addendum to registries for evaluating patient outcomes: a user’s guide. 3rd ed. Rockville, MD: US Agency for Healthcare Research and Quality, 2018. 29671989

[20] University of York, Centre for Reviews and Dissemination . 2009. Systematic reviews: CRD’s guidance for undertaking reviews in health care. Available at: https://www.york.ac.uk/media/crd/Systematic_Reviews.pdf. Accessed 17 December 2024.

[21] Downs SH, Black N. The feasibility of creating a checklist for the assessment of the methodological quality both of randomised and non-randomised studies of health care interventions. J Epidemiol Community Health 1998; 52:377–84. 9764259 10.1136/jech.52.6.377 PMC1756728

[22] Higgins JP, Thompson SG, Deeks JJ, Altman DG. Measuring inconsistency in meta-analyses. BMJ 2003; 327:557–60. 12958120 10.1136/bmj.327.7414.557 PMC192859

[23] Cochran WG . The combination of estimates from different experiments. Biometrics 1954; 10:101–29.

[24] Bechman K, Yates M, Mann K, et al Inpatient COVID-19 mortality has reduced over time: results from an observational cohort. PLoS One 2022; 17:e0261142. 35025917 10.1371/journal.pone.0261142 PMC8757902

[25] Beigel JH, Tomashek KM, Dodd LE, et al Remdesivir for the treatment of Covid-19—final report. N Engl J Med 2020; 383:1813–26. 32445440 10.1056/NEJMoa2007764 PMC7262788

[26] Benfield T, Bodilsen J, Brieghel C, et al Improved survival among hospitalized patients with coronavirus disease 2019 (COVID-19) treated with remdesivir and dexamethasone. A nationwide population-based cohort study. Clin Infect Dis 2021; 73:2031–6. 34111274 10.1093/cid/ciab536 PMC8344480

[27] Breskin A, Wiener C, Adimora AA, et al Effectiveness of remdesivir treatment protocols among patients hospitalized with COVID-19: a target trial emulation. Epidemiology 2023; 34:365–75. 36719738 10.1097/EDE.0000000000001598

[28] Caffrey AR, Liao JX, Lopes VV, LaPlante KL, Appaneal HJ. Real-world safety and effectiveness of remdesivir and corticosteroids in hospitalized patients with COVID-19. Covid 2023; 3:198–217.

[29] Chokkalingam AP, Hayden J, Goldman JD, et al Association of remdesivir treatment with mortality among hospitalized adults with COVID-19 in the United States. JAMA Netw Open 2022; 5:e2244505. 36454570 10.1001/jamanetworkopen.2022.44505 PMC9716380

[30] De Vito A, Poliseno M, Zauli B, et al Efficacy of remdesivir in reducing mortality in people hospitalised for SARS-CoV-2 infection: a real-life experience. Presented at: ECCMID 2022 April 23–26, Lisbon, Portugal.

[31] Diaz GA, Christensen AB, Pusch T, et al Remdesivir and mortality in patients with coronavirus disease 2019. Clin Infect Dis 2022; 74:1812–20. 34409431 10.1093/cid/ciab698 PMC9155603

[32] Dobrowolska K, Zarebska-Michaluk D, Brzdek M, et al Retrospective analysis of the effectiveness of remdesivir in COVID-19 treatment during periods dominated by Delta and Omicron SARS-CoV-2 variants in clinical settings. J Clin Med 2023; 12:19. 10.3390/jcm12062371 PMC10051185 36983370

[33] Finn A, Jindal A, Andrea SB, Selvaraj V, Dapaah-Afriyie K. Association of treatment with remdesivir and 30-day hospital readmissions in patients hospitalized with COVID-19: multicenter study. Am J Med Sci 2022; 363:403–10. 35151637 10.1016/j.amjms.2022.01.021 PMC8830144

[34] Garibaldi BT, Wang K, Robinson ML, et al Real-world effectiveness of remdesivir in adults hospitalized with coronavirus disease 2019 (COVID-19): a retrospective, multicenter comparative effectiveness study. Clin Infect Dis 2022; 75:e516–24. 34910128 10.1093/cid/ciab1035 PMC8754724

[35] Grundmann A, Wu CH, Hardwick M, et al Fewer COVID-19 neurological complications with dexamethasone and remdesivir. Ann Neurol 2023; 93:88–102. 36261315 10.1002/ana.26536 PMC9874556

[36] Lapadula G, Bernasconi DP, Bellani G, et al Remdesivir use in patients requiring mechanical ventilation due to COVID-19. Open Forum Infect Dis 2020; 7:ofaa481. 33204761 10.1093/ofid/ofaa481 PMC7651598

[37] Leding C, Bodilsen J, Brieghel C, et al Treatment effect modifiers in hospitalised patients with COVID-19 receiving remdesivir and dexamethasone. Infect Dis 2023; 55:351–60. 10.1080/23744235.2023.2187081 36905638

[38] Marx K, Goncarova K, Fedders D, et al Clinical outcomes of hospitalized COVID-19 patients treated with remdesivir: a retrospective analysis of a large tertiary care center in Germany. Infection 2023; 51:97–108. 35553032 10.1007/s15010-022-01841-8 PMC9098143

[39] Mozaffari E, Chandak A, Zhang Z, et al Remdesivir treatment in hospitalized patients with coronavirus disease 2019 (COVID-19): a comparative analysis of in-hospital all-cause mortality in a large multicenter observational cohort. Clin Infect Dis 2022; 75:e450–8. 34596223 10.1093/cid/ciab875 PMC9402660

[40] Mozaffari E, Chandak A, Gottlieb RL, et al Remdesivir reduces mortality in hospitalized covid-19 patients across variant eras. Conference abstract. Top Antivir Med 2023; 31:218–9.

[41] Olender SA, Walunas TL, Martinez E, et al Remdesivir versus standard-of-care for severe coronavirus disease 2019 infection: an analysis of 28-day mortality. Open Forum Infect Dis 2021; 8:ofab278. 34282406 10.1093/ofid/ofab278 PMC8244650

[42] Henao-Restrepo AM, Pan H, Peto R, et al Remdesivir and three other drugs for hospitalised patients with COVID-19: final results of the WHO Solidarity randomised trial and updated meta-analyses. Lancet 2022; 399:1941–53. 35512728 10.1016/S0140-6736(22)00519-0 PMC9060606

[43] Wang Y, Zhang D, Du G, et al Remdesivir in adults with severe COVID-19: a randomised, double-blind, placebo-controlled, multicentre trial. Lancet (London, England) 2020; 395:1569–78. 32423584 10.1016/S0140-6736(20)31022-9 PMC7190303

[44] Burns PB, Rohrich RJ, Chung KC. The levels of evidence and their role in evidence-based medicine. Plast Reconstr Surg 2011; 128:305–10. 21701348 10.1097/PRS.0b013e318219c171 PMC3124652

[45] Burns KEA, Laird M, Stevenson J, et al Adherence of clinical practice guidelines for pharmacologic treatments of hospitalized patients with COVID-19 to trustworthy standards: a systematic review. JAMA Netw Open 2021; 4:e2136263. 34889948 10.1001/jamanetworkopen.2021.36263 PMC8665373

[46] Cokljat M, Cruz CV, Carrara VI, et al Comparison of WHO versus national COVID-19 therapeutic guidelines across the world: not exactly a perfect match. BMJ Glob Health 2024; 9:e014188. 10.1136/bmjgh-2023-014188 PMC11043689 38649182

[47] Griggs EP, Mitchell PK, Lazariu V, et al Clinical epidemiology and risk factors for critical outcomes among vaccinated and unvaccinated adults hospitalized with COVID-19-VISION network, 10 states, June 2021–March 2023. Clin Infect Dis 2024; 78:338–48. 37633258 10.1093/cid/ciad505 PMC11293024

[48] Kojima N, Adams K, Self WH, et al Changing severity and epidemiology of adults hospitalized with coronavirus disease 2019 (COVID-19) in the United States after introduction of COVID-19 vaccines, March 2021–August 2022. Clin Infect Dis 2023; 77:547–57. 37255285 10.1093/cid/ciad276 PMC10526883

[49] Montejano R, Soler-Carracedo A, Borobia AM, et al The current mismatch between COVID-19 clinical trial design and the evolving profile of hospitalized patients: a retrospective cohort analysis. Clin Infect Dis 2024; 78:918–21. 37882613 10.1093/cid/ciad655

[50] Fawzy A, Wu TD, Wang K, et al Racial and ethnic discrepancy in pulse oximetry and delayed identification of treatment eligibility among patients with COVID-19. JAMA Intern Med 2022; 182:730–8. 35639368 10.1001/jamainternmed.2022.1906 PMC9257583

[51] Magesh S, John D, Li WT, et al Disparities in COVID-19 outcomes by race, ethnicity, and socioeconomic status: a systematic review and meta-analysis. JAMA Netw Open 2021; 4:e2134147. 34762110 10.1001/jamanetworkopen.2021.34147 PMC8586903

[52] Mozaffari E, Chandak A, Amin AN, et al Racial and ethnic disparities in COVID-19 treatments in the United States. J Racial Ethn Health Disparities 2025; 12:1052–62. 38409487 10.1007/s40615-024-01942-0 PMC11914345

[53] Wiltz JL, Feehan AK, Molinari NM, et al Racial and ethnic disparities in receipt of medications for treatment of COVID-19—United States, March 2020–August 2021. MMWR Morb Mortal Wkly Rep 2022; 71:96–102. 35051133 10.15585/mmwr.mm7103e1 PMC8774154

[54] Bun RS, Scheer J, Guillo S, Tubach F, Dechartres A. Meta-analyses frequently pooled different study types together: a meta-epidemiological study. J Clin Epidemiol 2020; 118:18–28. 31698062 10.1016/j.jclinepi.2019.10.013

