# Supplementary material for: A Systematic Review and Meta-analysis of the Effectiveness of Remdesivir to Treat SARS-CoV-2 in Hospitalized Patients: Have the Guidelines Evolved With the Evidence?
Source: Clin Infect Dis. 2025 Mar 11;81(1):20–9. doi: 10.1093/cid/ciaf111 (PMC12314504; doi:10.1093/cid/ciaf111)
Supplement: ciaf111_Supplementary_Data [file ciaf111_supplementary_data.pdf]

Supplemental Material

Supplemental Methods

Due to heterogeneity in reporting outcomes across various study types we grouped studies into categories with similar characteristics. Results for mild-moderate disease were grouped with those for patients not requiring oxygen support at admission. Similarly, results for severe disease were grouped with those reported for patients requiring non-invasive ventilation, if low flow or high flow oxygen use was reported, and critical disease results were grouped with those reported for patients requiring invasive mechanical ventilation.

A hierarchical random-effects meta-analysis model was used to analyse the odds ratio (OR) for the binary endpoints, or the mean difference (MD) for the continuous endpoints. The model for the log OR / for the MD is

$$y_i \sim N(\theta_i, \sigma_i^2), \ \ \sigma_i^2 \text{ is known}$$

where i is the index for each study, y_i is the log OR / the MD of remdesivir vs. control, and σ_i is the standard error of the log OR / the MD. The expected log OR / the expected MD is

$$\theta_i = \theta + u_i, \text{ with } u_i \sim N(0, \tau_{k(i)}^2)$$

where θ is the averaged effect of remdesivir across studies; the u_i are study-level random effects that represent residual variance from the study i. This base model estimates the variance of the random effects by study $\tau_{k(j)}^2$, which is nested by

study type k. For example, the variance could differ by study type (i.e., RW studies [RWS]/RCT) and may be smaller for RCTs compared to RWSs due to RCTs attempting to reduce variation as a design goal in many cases.

In a likelihood-based inference framework, the parameters to be estimated are the averaged effect θ, and the random effects standard deviations τ_{RCT} and τ_{RWS}.

First, we conducted a meta-analysis for the overall population of hospitalized patients with SARS-CoV-2 infection (i.e., not stratified by the oxygen support requirement at admission, an indicator of the patients' disease severity). In a subsequent step, the weighted average across four levels of oxygen support (i.e., no supplemental oxygen (NSO), low flow oxygen (LFO), high flow oxygen (HFO) and invasive mechanical ventilation/extracorporeal membrane oxygenation (IMV/ ECMO) was incorporated in the meta-analysis. Pooled results were adjusted for the duration of remdesivir treatment and the proportion of the population requiring oxygen support at baseline.

Supplemental Table S1. Summary of guideline recommendations for remdesivir use in hospitalized patients with COVID-19 in the general population and the scientific evidence base

GUIDELINE (date of last update for RDV recommendations)	Recommendation	Scientific basis for recommendations (trial name or author)	Year of publication	Study design	Study period
**National Institutes of Health (NIH), US[1] (February 2024)**	➢ For the following hospitalized patient categories, RDV is recommended to be administered for 5 days (or	ACTT-1<br>CATCO<br>DisCoVeRy<br>Goldman, et al.	2020<br>2022<br>2021<br>2020	RCT<br>RCT<br>RCT<br>RCT	Feb 2020 - May 2020<br>Aug 2020 - Apr 2021<br>Mar 2020 - Jan 2021<br>Mar 2020 - Jun 2020

GUIDELINE (date of last update for RDV recommendations)	Recommendation	Scientific basis for recommendations (trial name or author)	Year of publication	Study design	Study period
	until hospital discharge, whichever comes first):<br>• **LFO**<br>o minimal conventional oxygen<br>• **HFNC/NIV**<br>o all patients who:<br>▪ are immunocompromised<br>▪ have evidence of ongoing viral replication<br>▪ are within 10 days of symptom onset.<br>➢ There is **insufficient evidence** for the Panel to recommend either for or against RDV use in patients requiring **IMV/ECMO**.	Mozaffari, et al.<br>PINETREE<br>REMDACTA<br>Spinner, et al.<br>Wang, et al.<br>WHO Solidarity Trial, Final Report	2023<br>2021<br>2021<br>2020<br>2020<br>2022	RWD<br>RCT<br>RCT<br>RCT<br>RCT<br>RCT	Dec 2020 - Apr 2022<br>Sep 2020 - May 2021<br>Jun 2020 -Jan 2021<br>Mar 2020 - May 2020<br>Mar 2020 - May 2020<br>Mar 2020 - Jan 2021
**European Society of Clinical Microbiology and Infectious Diseases (ESCMID)[2, 3] (August 2022)**	➢ There is a **conditional recommendation** for RDV use in patients with mild COVID-19.	ACTT-1<br>Budi, et al.	2020<br>2022	RCT<br>SR based on 13 observation studies with 113 pregnant people	Feb 2020 - May 2020<br>The search was conducted on July 26, 2021
	➢ There is a **conditional recommendation against** RDV use in patients with severe/critical COVID-19 requiring HFO.	Mahajan, et al.<br>Mozaffari, et al.<br>Spinner, et al.<br>Wang, et al.	2021<br>2021<br>2020<br>2020	RCT<br>RWD<br>RCT<br>RCT	Jun 2020 - Dec 2020<br>Aug 2020 - Nov 2020<br>Mar 2020 - May 2020<br>Feb 2020 - Mar 2020
	➢ There is a **conditional recommendation** for RDV use in hospitalized COVID-19				

GUIDELINE (date of last update for RDV recommendations)	Recommendation	Scientific basis for recommendations (trial name or author)	Year of publication	Study design	Study period
	patients not requiring **IMV/ECMO**.				
**Infectious Diseases Society of America (IDSA), US[4] (July 2022)**	➢ There is a **conditional recommendation** for a 5-day RDV course (rather than 10-day) in patients **on supplemental oxygen not mechanically ventilated**.<br>➢ **In severe COVID-19** patients RDV treatment is **suggested over no antiviral** treatment.<br>➢ **There is a recommendation against routine initiation of RDV in patients on IMV/ECMO.**	ACTT-1	2020	RCT	Feb 2020 - May 2020
		Goldman, et al.	2020	RCT	Mar 2020 - Jun 2020
		PINETREE	2021	RCT	Sep 2020 - May 2021
		Wang, et al.	2020	RCT	Feb 2020 - Mar 2020
		WHO Solidarity Trial, Final Report	2022	RCT	Mar 2020 - Jan 2021
**World Health Organization (WHO)[5] (September 2022)**	➢ **Conditional recommendation** for RDV use in patients with **severe COVID-19.**<br>➢ **Conditional recommendation** against RDV use in patients with **critical COVID-19.**	ACTT-1	2020	RCT	Feb 2020 - May 2020
		CATCO	2022	RCT	Aug 2020 - Apr 2021
		DisCoVeRy	2021	RCT	Mar 2020 - Jan 2021
		Mahajan, et al.	2021	RCT	Jun 2020 - Dec 2020
		PINETREE	2021	RCT	Sep 2020 - May 2021
		Wang, et al.	2020	RCT	Feb 2020 - Mar 2020
		WHO Solidarity Trial, Final Report	2022	RCT	Mar 2020 - Jan 2021
**European Respiratory Society[6-8] (August 2022)**	➢ **No recommendation** for use of RDV in patients **not requiring IMV.**<br>➢ Recommendation **against RDV** use in patients **requiring IMV.**	ACTT-1	2020	RCT	Feb 2020 - May 2020
		Crichton, et al.	2020	SR of CT	Search up until the end of February 2021
		Mahajan, et al.	2021	RCT	Jun 2020 - Dec 2020
		PINETREE	2021	RCT	Sep 2020 - May 2021
		Spinner, et al.	2020	RCT	Mar 2020 - May 2020
		Wang, et al.	2020	RCT	Feb 2020 - Mar 2020

GUIDELINE (date of last update for RDV recommendations)	Recommendation	Scientific basis for recommendations (trial name or author)	Year of publication	Study design	Study period
		WHO Solidarity Trial, Final Report	2022	RCT	Mar 2020 - Jan 2021

ECMO, extracorporeal membrane oxygenation; HFNC, high flow nasal cannula; IMV, invasive mechanical ventilation; LFO, low flow oxygen; MV, mechanical ventilation; NIV, non-invasive mechanical ventilation; NSO, no supplemental oxygen; RCT, randomized controlled trial; RDV, remdesivir; RWD, real world data; SR, systematic review.

Supplemental Table S2. **Search terms for use in MEDLINE**

Term Group	#	Search String
**COVID-19**	1	Coronavirus/ or Coronavirus Infections/
	2	SARS-CoV-2.ti,ab,kf.
	3	(coronavirus$ or corona virus$).ti,ab,kf.
	4	(Covid-19 or Covid19).ti,ab,kf.
	5	nCoV.ti,ab,kf.
	6	or/1-5
**RCTs**	7	Randomized Controlled Trials as Topic/
	8	Randomized Controlled Trial/
	9	Random Allocation/
	10	Double-Blind Method/
	11	Single-Blind Method/
	12	Placebos/
	13	exp Clinical Trials as Topic/
	14	Clinical Trial/
	15	Clinical Trial, Phase II/ or Clinical Trial, Phase III/ or Clinical Trial, Phase IV/
	16	Controlled Clinical Trial/ or Adaptive Clinical Trial/
	17	randomized controlled trial.pt.
	18	clinical trial.pt.
	19	(clinical trial, phase ii or clinical trial, phase iii or clinical trial, phase iv).pt.
	20	(controlled clinical trial or multicenter study).pt.
	21	(clinical adj trial$).ti,ab,kf.
	22	((singl$ or doubl$ or treb$ or tripl$) adj (blind$3 or mask$3)).ti,ab,kf.
	23	placebo$.ti,ab,kf.
	24	(allocat$ adj2 random$).ti,ab,kf.
	25	Randomi?ed adj2 trial$.ti,ab,kf.
	26	rct.ti,ab,kf.
	27	or/7-26
**Interventional non-RCTs and Observational Studies**	28	Clinical Trial, Phase I/
	29	clinical trial, phase i.pt.
	30	(single arm adj3 (trial$ or stud$)).ti,ab,kf.
	31	(open label adj (trial$ or stud$)).ti,ab,kf.
	32	(non blinded adj (trial$ or stud$)).ti,ab,kf.
	33	(pragmatic trial$ or pragmatic stud$).ti,ab,kf. or Pragmatic Clinical Trial/
	34	Epidemiologic Studies/
	35	Observational Study/
	36	Cohort Studies/
	37	exp Case-Control Studies/
	38	Cross-Sectional Studies/
	39	Clinical Study/

	40	Follow-Up Studies/
	41	Longitudinal Studies/
	42	Retrospective Studies/
	43	Prospective Studies/ not Randomized Controlled Trials as Topic/
	44	(observational adj (study or studies)).ti,ab,kf.
	45	(cohort adj (study or studies)).ti,ab,kf.
	46	cohort analy$.ti,ab,kf.
	47	case control.ti,ab,kf.
	48	cross sectional.ti,ab,kf.
	49	(follow up adj (study or studies)).ti,ab,kf.
	50	longitudinal.ti,ab,kf.
	51	retrospective.ti,ab,kf.
	52	(chart adj3 review$).ti,ab,kf.
	53	exp Registries/
	54	(registry or registries).ti,ab,kf.
	55	(prospective adj (study or studies)).ti,ab,kf.
	56	(epidemiologic$ adj (study or studies)).ti,ab,kf.
	57	(evaluation adj (study or studies)).ti,ab,kf.
	58	(medical record$ or real world or population based or survey$ or questionnaire$ or medicare or medicaid or marketscan).ti,ab,kf.
	59	or/28-58
**Remdesivir**	60	(remdesivir or RDV or veklury or GS-5734 or GS5734 or redyx).af.
**Exclusion Terms**	61	exp animals/ not exp humans/
	62	(comment or editorial or "case reports" or "historical article").pt.
	63	(case stud$ or case report$).ti.
	64	or/61-63
**Combination**	65	6 and (27 or 59) and 60
	66	65 not 64
	67	limit 66 to yr="2019-current"
	68	remove duplicates from 67

Databases: Ovid MEDLINE(R) and Epub Ahead of Print, In-Process, In-Data-Review & Other Non-Indexed Citations and Daily 1946 to December 5th, 2023

<u>Supplemental Table S3.</u> Search terms for use in Embase

Term Group	#	Search String
**COVID-19**	1	coronaviridae/ or coronavirus infection/
	2	SARS-CoV-2.ti,ab,kf.
	3	(coronavirus$ or corona virus$).ti,ab,kf.
	4	(Covid-19 or Covid19).ti,ab,kf.
	5	nCoV.ti,ab,kf.
	6	or/1-5
**RCTs**	7	"randomized controlled trial (topic)"/
	8	randomized controlled trial/
	9	randomization/
	10	double blind procedure/
	11	single blind procedure/
	12	crossover procedure/
	13	placebo/
	14	exp "clinical trial (topic)"/
	15	clinical trial/
	16	phase 2 clinical trial/ or phase 3 clinical trial/ or phase 4 clinical trial/
	17	controlled clinical trial/ or adaptive clinical trial/ or multicenter study/
	18	(clinical adj trial$).ti,ab,kf.
	19	((singl$ or doubl$ or treb$ or tripl$) adj (blind$3 or mask$3)).ti,ab,kf.
	20	placebo$.ti,ab,kf.
	21	(allocat$ adj2 random$).ti,ab,kf.
	22	Randomi?ed adj2 trial$.ti,ab,kf.
	23	rct.ti,ab,kf.
	24	or/7-23
**Interventional non-RCTs and Observational Studies**	25	phase 1 clinical trial/
	26	(clinical trial, phase i).pt.
	27	(single arm adj3 (trial$ or stud$)).ti,ab,kf.
	28	(open label adj (trial$ or stud$)).ti,ab,kf.
	29	(non blinded adj (trial$ or stud$)).ti,ab,kf.
	30	(pragmatic trial$ or pragmatic stud$).ti,ab,kf. or pragmatic trial/
	31	observational study/
	32	cohort analysis/
	33	exp case control study/
	34	cross-sectional study/
	35	clinical study/
	36	follow up/
	37	longitudinal study/
	38	retrospective study/
	39	prospective study/ not "randomized controlled trial (topic)"/

	40	(observational adj (study or studies)).ti,ab,kf.
	41	(cohort adj (study or studies)).ti,ab,kf.
	42	cohort analy$.ti,ab,kf.
	43	case control.ti,ab,kf.
	44	cross sectional.ti,ab,kf.
	45	(follow up adj (study or studies)).ti,ab,kf.
	46	longitudinal.ti,ab,kf.
	47	retrospective.ti,ab,kf.
	48	(chart adj3 review$).ti,ab,kf.
	49	exp disease registry/
	50	(registry or registries).ti,ab,kf.
	51	(prospective adj (study or studies)).ti,ab,kf.
	52	(epidemiologic$ adj (study or studies)).ti,ab,kf.
	53	(evaluation adj (study or studies)).ti,ab,kf.
	54	(medical record$ or real world or population based or survey$ or questionnaire$ or medicare or medicaid or marketscan).ti,ab,kf.
	55	or/25-54
**Remdesivir**	56	exp remdesivir/ or (remdesivir or RDV or veklury or GS-5734 or GS5734 or redyx).af.
**Exclusion Terms**	57	exp animals/ not exp humans/
	58	editorial.pt.
	59	editorial/ or case report/
	60	(case stud$ or case report$).ti.
	61	or/57-60
**Combination**	62	6 and (24 or 55) and 56
	63	62 not 61
	64	limit 63 to yr="2019-current"
	65	remove duplicates from 64

Database: Embase 1974 to December 5th, 2023

Supplemental Table S4. Search terms used in the Cochrane Library databases

Term Group	#	Search String
**COVID-19**	1	[mh "Coronavirus"]
	2	[mh "Coronavirus Infections"]
	3	(SARS-CoV-2):ab,ti,kw
	4	(coronavirus* or (corona virus*)):ab,ti,kw
	5	(Covid-19 or Covid19):ab,ti,kw
	6	(nCoV):ab,ti,kw
	7	{OR #1-#6}
**Remdesivir**	8	(remdesivir or RDV or veklury or GS-5734 or GS5734 or redyx):ab,ti,kw
**Combination**	9	#7 AND #8
	10	#9 with Publication Year from 2019 to present, in Trials
	11	#9 with Cochrane Library publication date from 2019 to present, in Cochrane Reviews

Supplemental Table S5. SLR eligibility criteria

CATEGORY	INCLUSION CRITERIA	EXCLUSION CRITERIA
**Patient population**	Unselected[a] adult or adolescent patients (aged ≥12 years) hospitalized with COVID-19	• Animal, *in vitro* or *in silico* studies<br>• Selected populations (e.g., pregnant women or patients with cancer) or children <12 years of age<br>• Patients without COVID-19<br>• Non-hospitalized patients
**Intervention[b]**	Therapy as monotherapy or in combination with SoC:<br>• Remdesivir	• Any other intervention
**Comparator**	Any	N/A

CATEGORY	INCLUSION CRITERIA	EXCLUSION CRITERIA
**Outcomes**	Clinical efficacy outcomes, including but not limited to:<br>• Mortality rate<br>• Length of hospital stay/ time to discharge<br>• Change in ordinal scales<br>• Normalisation or reduction of fever<br>• Need for NIV<br>• Need for supplemental oxygen<br>• Clinical recovery/improvement<br>• Respiratory progression<br>• Viral load reduction<br>• Change in severity<br>• System-specific complications<br>Safety outcomes e.g., treatment-emergent adverse events	• Studies not presenting relevant outcomes for the population of interest
**Study design**	Any of the following:<br>• RCTs<br>• Interventional non-RCTs<br>• Single-arm clinical trials<br>• Observational studies<br>• Real-world studies<br>• Protocols or trial records if they report results or are associated with publications reporting results[b]	• Any other type of analysis<br>• Case reports<br>• Case series
	Relevant SLRs and [N]MAs were included at the title/abstract review stage and were hand-searched for primary studies.	
**Publication type**	• Primary research articles<br>• Letters, if they reported primary research<br>• Congress abstracts published since 2020	• Any other publication type, including studies not reporting any original research<br>• Congress abstracts published before 2020
**Language**	Any language[b]	N/A
**Other**	Any country	N/A

a Unselected - trials that do not limit to specific populations.

b Database searches were limited to English sources, but any non-English relevant records identified from the searches were included.

ARDS, acute respiratory distress syndrome; N/A, not applicable; [N]MA, (network) meta-analysis; RCT, randomized controlled clinical trial; SLR, systematic literature review; SoC, standard of care.

<u>Supplemental Table S6</u>. Risk of bias assessment of RCTs by criteria provided by the York Centre for Reviews and Dissemination

Study name	Randomization	Allocation concealment	Baseline characteristics	Blinding	Withdrawals	Outcome selection and reporting	Statistical methodology
ACTT-1[9]	Low risk	Low risk	Unclear	Low risk	Low risk	Low risk	Low risk
ACTT-2[10]	Low risk	Unclear	Unclear	Unclear	Low risk	Low risk	Low risk
ACTT-3[11]	Low risk	Unclear	Unclear	Low risk	Low risk	Low risk	Low risk
ACTT-4[12]	Low risk	Unclear	Unclear	Low risk	Low risk	Low risk	Low risk
CATCO[13]	Low risk	Unclear	Low risk	High risk	High risk	High risk	Low risk
DisCoVeRy[14]	Low risk	Low risk	Low risk	High risk	Low risk	Unclear	Low risk
Hormati 2023[15]	Unclear	Unclear	Low risk	High risk	Low risk	High risk	Low risk
ITAC (INSIGHT 013)[16]	Low risk	Low risk	High risk	Low risk	Low risk	Low risk	High risk
LIVE-AIR[17]	Low risk	Low risk	Low risk	Low risk	Low risk	Low risk	Low risk
Mahajan 2021[18]	Low risk	Unclear	High risk	High risk	High risk	High risk	High risk
NOR-Solidarity[19]	Low risk	Unclear	Low risk	High risk	Low risk	Low risk	Low risk
PLATCOV[20]	Low risk	High risk	Low risk	High risk	Low risk	Low risk	High risk
REMDACTA[21]	Low risk	Unclear	Low risk	Low risk	Low risk	Low risk	High risk
SIMPLE-Moderate[22]	Low risk	Low risk	Low risk	High risk	High risk	High risk	High risk
SIMPLE-Severe[23]	Unclear	Unclear	High risk	High risk	High risk	Low risk	High risk
Solidarity[17]	Unclear	Unclear	Low risk	High risk	High risk	Low risk	Low risk
Solidarity Finland[18]	Unclear	High risk	Unclear	High risk	Low risk	Low risk	Low risk
Wang 2020[24]	Low risk	Low risk	High risk	Low risk	Low risk	Unclear	Low risk

Supplemental Table S7. Risk of bias assessment of observational studies by the Down's and Black Checklist

Author, year	Reporting	External validity	Confounding	Power
**Acharya 2022**[25]	High risk: 2/9<br>Low risk: 6/9<br>Unclear: 1/9	High risk: 0/3<br>Low risk: 3/3<br>Unclear: 0/3	High risk: 6/13<br>Low risk: 4/13<br>Unclear: 3/13	Unclear
**Aiello 2023**[26]	High risk: 5/9<br>Low risk: 3/9<br>Unclear: 1/9	High risk: 0/3<br>Low risk: 1/3<br>Unclear: 2/3	High risk: 5/13<br>Low risk: 5/13<br>Unclear: 3/13	Unclear
**Aksak-Was 2022**[27]	High risk: 3/9<br>Low risk: 6/9<br>Unclear: 0/9	High risk: 0/3<br>Low risk: 1/3<br>Unclear: 2/3	High risk: 5/13<br>Low risk: 5/13<br>Unclear: 3/13	Unclear
**Alexander 2023**[28]	High risk: 2/9<br>Low risk: 5/9<br>Unclear: 2/9	High risk: 0/3<br>Low risk: 3/3<br>Unclear: 0/3	High risk: 6/13<br>Low risk: 5/13<br>Unclear: 2/13	Unclear
**Alshamrani 2023**[29]	High risk: 3/9<br>Low risk: 6/9<br>Unclear: 0/9	High risk: 0/3<br>Low risk: 3/3<br>Unclear: 0/3	High risk: 5/13<br>Low risk: 5/13<br>Unclear: 3/13	Unclear
**Arribas López 2023**[30]	High risk: 2/9<br>Low risk: 6/9<br>Unclear: 1/9	High risk: 0/3<br>Low risk: 1/3<br>Unclear: 2/3	High risk: 5/13<br>Low risk: 7/13<br>Unclear: 1/13	Unclear
**Attena 2023**[31]	High risk: 2/9<br>Low risk: 7/9<br>Unclear: 0/9	High risk: 0/3<br>Low risk: 3/3<br>Unclear: 0/3	High risk: 5/13<br>Low risk: 6/13<br>Unclear: 2/13	Unclear
**Ayodele 2021**[32]	High risk: 4/9<br>Low risk: 5/9<br>Unclear: 0/9	High risk: 1/3<br>Low risk: 1/3<br>Unclear: 1/3	High risk: 7/13<br>Low risk: 4/13<br>Unclear: 2/13	Unclear
**Bansal 2021**[33]	High risk: 3/9<br>Low risk: 4/9<br>Unclear: 2/9	High risk: 0/3<br>Low risk: 3/3<br>Unclear: 0/3	High risk: 6/13<br>Low risk: 4/13<br>Unclear: 3/13	Unclear
**Bárczi 2023**[34]	High risk: 5/9<br>Low risk: 2/9<br>Unclear: 2/9	High risk: 1/3<br>Low risk: 0/3<br>Unclear: 2/3	High risk: 8/13<br>Low risk: 3/13<br>Unclear: 2/13	Unclear
**Basoulis 2023**[35]	High risk: 5/9<br>Low risk: 3/9<br>Unclear: 1/9	High risk: 2/3<br>Low risk: 1/3<br>Unclear: 0/3	High risk: 6/13<br>Low risk: 3/13<br>Unclear: 4/13	Unclear
**Bavaro 2023**[36]	High risk: 4/9<br>Low risk: 5/9<br>Unclear: 0/9	High risk: 0/3<br>Low risk: 3/3<br>Unclear: 0/3	High risk: 5/13<br>Low risk: 6/13<br>Unclear: 2/13	Unclear
**Bechman 2022**[37]	High risk: 3/9<br>Low risk: 5/9<br>Unclear: 1/9	High risk: 0/3<br>Low risk: 1/3<br>Unclear: 2/3	High risk: 6/13<br>Low risk: 4/13<br>Unclear: 3/13	Unclear
**Behboodikhah 2022**[38]	High risk: 5/9<br>Low risk: 2/9<br>Unclear: 2/9	High risk: 0/3<br>Low risk: 1/3<br>Unclear: 2/3	High risk: 7/13<br>Low risk: 3/13<br>Unclear: 3/13	Unclear
**Benfield 2021**[39]	High risk: 4/9<br>Low risk: 2/9<br>Unclear: 3/9	High risk: 0/3<br>Low risk: 3/3<br>Unclear: 0/3	High risk: 7/13<br>Low risk: 2/13<br>Unclear: 4/13	Unclear
**Bernal 2023**[40]	High risk: 4/9<br>Low risk: 4/9<br>Unclear: 1/9	High risk: 0/3<br>Low risk: 3/3<br>Unclear: 0/3	High risk: 6/13<br>Low risk: 5/13<br>Unclear: 2/13	Unclear
**Bistrovic 2022**[41]	High risk: 5/9<br>Low risk: 1/9	High risk: 1/3<br>Low risk: 2/3	High risk: 5/13<br>Low risk: 5/13	Unclear

Author, year	Reporting	External validity	Confounding	Power
	Unclear: 3/9	Unclear: 0/3	Unclear: 3/13	
**Boglione 2022**[42]	High risk: 1/9<br>Low risk: 5/9<br>Unclear: 3/9	High risk: 0/3<br>Low risk: 3/3<br>Unclear: 0/3	High risk: 6/13<br>Low risk: 4/13<br>Unclear: 3/13	Unclear
**Breskin 2023**[43]	High risk: 3/9<br>Low risk: 6/9<br>Unclear: 0/9	High risk: 1/3<br>Low risk: 2/3<br>Unclear: 0/3	High risk: 6/13<br>Low risk: 4/13<br>Unclear: 3/13	Unclear
**Burhan 2023**[44]	High risk: 2/9<br>Low risk: 6/9<br>Unclear: 1/9	High risk: 0/3<br>Low risk: 3/3<br>Unclear: 0/3	High risk: 7/13<br>Low risk: 2/13<br>Unclear: 4/13	Unclear
**Butt 2023**[45]	High risk: 2/9<br>Low risk: 5/9<br>Unclear: 2/9	High risk: 1/3<br>Low risk: 2/3<br>Unclear: 0/3	High risk: 5/13<br>Low risk: 6/13<br>Unclear: 2/13	Unclear
**Caffrey 2021**[46]	High risk: 7/9<br>Low risk: 0/9<br>Unclear: 2/9	High risk: 1/3<br>Low risk: 0/3<br>Unclear: 2/3	High risk: 6/13<br>Low risk: 2/13<br>Unclear: 5/13	Unclear
**Caffrey 2023**[47]	High risk: 1/9<br>Low risk: 5/9<br>Unclear: 3/9	High risk: 0/3<br>Low risk: 3/3<br>Unclear: 0/3	High risk: 5/13<br>Low risk: 5/13<br>Unclear: 3/13	Unclear
**CARAVAN**[48]	High risk: 1/9<br>Low risk: 8/9<br>Unclear: 0/9	High risk: 1/3<br>Low risk: 0/3<br>Unclear: 2/3	High risk: 5/13<br>Low risk: 4/13<br>Unclear: 4/13	Unclear
**Chaudhary 2023**[49]	High risk: 3/9<br>Low risk: 5/9<br>Unclear: 1/9	High risk: 0/3<br>Low risk: 3/3<br>Unclear: 0/3	High risk: 5/13<br>Low risk: 5/13<br>Unclear: 3/13	Unclear
**Chavalertsakul 2023**[50]	High risk: 4/9<br>Low risk: 3/9<br>Unclear: 2/9	High risk: 1/3<br>Low risk: 1/3<br>Unclear: 1/3	High risk: 7/13<br>Low risk: 2/13<br>Unclear: 4/13	Unclear
**Chokkalingam 2022**[51]	High risk: 2/9<br>Low risk: 6/9<br>Unclear: 1/9	High risk: 0/3<br>Low risk: 3/3<br>Unclear: 0/3	High risk: 5/13<br>Low risk: 5/13<br>Unclear: 3/13	Unclear
**Cilloniz 2023**[52]	High risk: 1/9<br>Low risk: 7/9<br>Unclear: 1/9	High risk: 0/3<br>Low risk: 3/3<br>Unclear: 0/3	High risk: 5/13<br>Low risk: 7/13<br>Unclear: 1/13	Unclear
**Das 2022**[53]	High risk: 7/9<br>Low risk: 1/9<br>Unclear: 1/9	High risk: 0/3<br>Low risk: 0/3<br>Unclear: 3/3	High risk: 6/13<br>Low risk: 4/13<br>Unclear: 3/13	Unclear
**De Vito 2022**[54]	High risk: 5/9<br>Low risk: 3/9<br>Unclear: 1/9	High risk: 0/3<br>Low risk: 3/3<br>Unclear: 0/3	High risk: 6/13<br>Low risk: 4/13<br>Unclear: 3/13	Unclear
**De Vito 2023**[55]	High risk: 3/9<br>Low risk: 4/9<br>Unclear: 2/9	High risk: 0/3<br>Low risk: 3/3<br>Unclear: 0/3	High risk: 5/13<br>Low risk: 5/13<br>Unclear: 3/13	Unclear
**Delgado 2023**[56]	High risk: 2/9<br>Low risk: 6/9<br>Unclear: 1/9	High risk: 1/3<br>Low risk: 0/3<br>Unclear: 2/3	High risk: 5/13<br>Low risk: 5/13<br>Unclear: 3/13	Unclear
**Devgun 2023**[57]	High risk: 2/9<br>Low risk: 6/9<br>Unclear: 1/9	High risk: 0/3<br>Low risk: 2/3<br>Unclear: 1/3	High risk: 6/13<br>Low risk: 4/13<br>Unclear: 3/13	Unclear
**Diaz 2022**[58]	High risk: 2/9<br>Low risk: 6/9<br>Unclear: 1/9	High risk: 0/3<br>Low risk: 3/3<br>Unclear: 0/3	High risk: 5/13<br>Low risk: 7/13<br>Unclear: 1/13	Unclear

Author, year	Reporting	External validity	Confounding	Power
**Dobrowolska 2023**[59]	High risk: 1/9<br>Low risk: 6/9<br>Unclear: 2/9	High risk: 1/3<br>Low risk: 0/3<br>Unclear: 2/3	High risk: 6/13<br>Low risk: 5/13<br>Unclear: 2/13	Unclear
**Elshaboury 2022**[60]	High risk: 5/9<br>Low risk: 3/9<br>Unclear: 1/9	High risk: 0/3<br>Low risk: 2/3<br>Unclear: 1/3	High risk: 6/13<br>Low risk: 4/13<br>Unclear: 3/13	Unclear
**Estill 2023**[61]	High risk: 3/9<br>Low risk: 6/9<br>Unclear: 0/9	High risk: 1/3<br>Low risk: 2/3<br>Unclear: 0/3	High risk: 5/13<br>Low risk: 6/13<br>Unclear: 2/13	Unclear
**Falcone 2022**[62]	High risk: 2/9<br>Low risk: 6/9<br>Unclear: 1/9	High risk: 0/3<br>Low risk: 3/3<br>Unclear: 0/3	High risk: 5/13<br>Low risk: 6/13<br>Unclear: 1/13	Unclear
**Finn 2022**[63]	High risk: 3/9<br>Low risk: 5/9<br>Unclear: 1/9	High risk: 1/3<br>Low risk: 1/3<br>Unclear: 1/3	High risk: 4/13<br>Low risk: 8/13<br>Unclear: 1/13	Unclear
**Garibaldi 2022**[64]	High risk: 3/9<br>Low risk: 5/9<br>Unclear: 1/9	High risk: 1/3<br>Low risk: 1/3<br>Unclear: 1/3	High risk: 5/13<br>Low risk: 7/13<br>Unclear: 1/13	Unclear
**Gragera Gomez 2022**[65]	High risk: 4/9<br>Low risk: 3/9<br>Unclear: 2/9	High risk: 1/3<br>Low risk: 1/3<br>Unclear: 1/3	High risk: 6/13<br>Low risk: 3/13<br>Unclear: 4/13	Unclear
**GREC**[66]	High risk: 1/9<br>Low risk: 7/9<br>Unclear: 1/9	High risk: 0/3<br>Low risk: 2/3<br>Unclear: 1/3	High risk: 5/13<br>Low risk: 4/13<br>Unclear: 4/13	Unclear
**Grundmann 2023**[67]	High risk: 4/9<br>Low risk: 5/9<br>Unclear: 0/9	High risk: 1/3<br>Low risk: 2/3<br>Unclear: 0/3	High risk: 4/13<br>Low risk: 6/13<br>Unclear: 3/13	Unclear
**Gunasekaran 2021**[68]	High risk: 5/9<br>Low risk: 3/9<br>Unclear: 1/9	High risk: 0/3<br>Low risk: 1/3<br>Unclear: 2/3	High risk: 5/13<br>Low risk: 2/13<br>Unclear: 6/13	Unclear
**Jeyapalina 2022**[69]	High risk: 5/9<br>Low risk: 2/9<br>Unclear: 2/9	High risk: 0/3<br>Low risk: 2/3<br>Unclear: 1/3	High risk: 5/13<br>Low risk: 5/13<br>Unclear: 3/13	Unclear
**Karolyi 2022**[70]	High risk: 4/9<br>Low risk: 4/9<br>Unclear: 1/9	High risk: 0/3<br>Low risk: 1/3<br>Unclear: 2/3	High risk: 6/13<br>Low risk: 2/13<br>Unclear: 5/13	Unclear
**Koh 2023**[71]	High risk: 2/9<br>Low risk: 6/9<br>Unclear: 1/9	High risk: 0/3<br>Low risk: 3/3<br>Unclear: 0/3	High risk: 5/13<br>Low risk: 5/13<br>Unclear: 3/13	High risk
**Kubiliute 2023**[72]	High risk: 3/9<br>Low risk: 4/9<br>Unclear: 2/9	High risk: 0/3<br>Low risk: 1/3<br>Unclear: 2/3	High risk: 6/13<br>Low risk: 4/13<br>Unclear: 3/13	Unclear
**Lakhanpal 2022**[73]	High risk: 2/9<br>Low risk: 5/9<br>Unclear: 2/9	High risk: 1/3<br>Low risk: 2/3<br>Unclear: 0/3	High risk: 6/13<br>Low risk: 5/13<br>Unclear: 2/13	Unclear
**Lapadula 2020**[74]	High risk: 2/9<br>Low risk: 7/9<br>Unclear: 0/9	High risk: 0/3<br>Low risk: 3/3<br>Unclear: 0/3	High risk: 5/13<br>Low risk: 6/13<br>Unclear: 2/13	Unclear
**Leding 2023**[75]	High risk: 2/9<br>Low risk: 5/9<br>Unclear: 2/9	High risk: 0/3<br>Low risk: 3/3<br>Unclear: 0/3	High risk: 5/13<br>Low risk: 5/13<br>Unclear: 3/13	Unclear
**Leegwater 2023**[76]	High risk: 2/9<br>Low risk: 6/9<br>Unclear: 1/9	High risk: 0/3<br>Low risk: 3/3<br>Unclear: 0/3	High risk: 5/13<br>Low risk: 5/13<br>Unclear: 3/13	Unclear

Author, year	Reporting	External validity	Confounding	Power
**Lim 2021**[77]	High risk: 3/9<br>Low risk: 6/9<br>Unclear: 0/9	High risk: 0/3<br>Low risk: 1/3<br>Unclear: 2/3	High risk: 6/13<br>Low risk: 5/13<br>Unclear: 2/13	Unclear
**Lim 2022**[78]	High risk: 2/9<br>Low risk: 7/9<br>Unclear: 0/9	High risk: 0/3<br>Low risk: 3/3<br>Unclear: 0/3	High risk: 5/13<br>Low risk: 6/13<br>Unclear: 2/13	High risk
**Lucijanic 2022**[79]	High risk: 2/9<br>Low risk: 6/9<br>Unclear: 1/9	High risk: 0/3<br>Low risk: 3/3<br>Unclear: 0/3	High risk: 5/13<br>Low risk: 5/13<br>Unclear: 3/13	Unclear
**Malik 2022**[80]	High risk: 3/9<br>Low risk: 5/9<br>Unclear: 1/9	High risk: 2/3<br>Low risk: 1/3<br>Unclear: 0/3	High risk: 5/13<br>Low risk: 6/13<br>Unclear: 2/13	Unclear
**Marocco 2023**[81]	High risk: 2/9<br>Low risk: 6/9<br>Unclear: 1/9	High risk: 0/3<br>Low risk: 3/3<br>Unclear: 0/3	High risk: 5/13<br>Low risk: 7/13<br>Unclear: 1/13	Unclear
**Marx 2022**[82]	High risk: 2/9<br>Low risk: 6/9<br>Unclear: 1/9	High risk: 1/3<br>Low risk: 2/3<br>Unclear: 0/3	High risk: 5/13<br>Low risk: 7/13<br>Unclear: 1/13	High risk
**Metchurtchlishvili 2023**[83]	High risk: 1/9<br>Low risk: 7/9<br>Unclear: 1/9	High risk: 0/3<br>Low risk: 3/3<br>Unclear: 0/3	High risk: 5/13<br>Low risk: 5/13<br>Unclear: 3/13	High risk
**Mohanty 2021**[84]	High risk: 2/9<br>Low risk: 7/9<br>Unclear: 0/9	High risk: 1/3<br>Low risk: 2/3<br>Unclear: 0/3	High risk: 7/13<br>Low risk: 4/13<br>Unclear: 2/13	Unclear
**Monardo 2022**[85]	High risk: 6/9<br>Low risk: 3/9<br>Unclear: 0/9	High risk: 0/3<br>Low risk: 2/3<br>Unclear: 1/3	High risk: 6/13<br>Low risk: 4/13<br>Unclear: 3/13	Unclear
**Mozaffari 2021a**[86]	High risk: 4/9<br>Low risk: 3/9<br>Unclear: 2/9	High risk: 0/3<br>Low risk: 1/3<br>Unclear: 2/3	High risk: 5/13<br>Low risk: 6/13<br>Unclear: 2/13	Unclear
**Mozaffari 2021b**[87]	High risk: 5/9<br>Low risk: 4/9<br>Unclear: 0/9	High risk: 0/3<br>Low risk: 3/3<br>Unclear: 0/3	High risk: 5/13<br>Low risk: 5/13<br>Unclear: 3/13	Unclear
**Mozaffari 2022**[88]	High risk: 3/9<br>Low risk: 6/9<br>Unclear: 0/9	High risk: 0/3<br>Low risk: 3/3<br>Unclear: 0/3	High risk: 5/13<br>Low risk: 5/13<br>Unclear: 3/13	Unclear
**Mozaffari 2023a**[89]	High risk: 2/9<br>Low risk: 6/9<br>Unclear: 1/9	High risk: 0/3<br>Low risk: 1/3<br>Unclear: 2/3	High risk: 5/13<br>Low risk: 5/13<br>Unclear: 3/13	Unclear
**Mozaffari 2023b**[90]	High risk: 3/9<br>Low risk: 4/9<br>Unclear: 2/9	High risk: 0/3<br>Low risk: 1/3<br>Unclear: 2/3	High risk: 5/13<br>Low risk: 5/13<br>Unclear: 3/13	Unclear
**Ngo 2022**[91]	High risk: 3/9<br>Low risk: 6/9<br>Unclear: 0/9	High risk: 0/3<br>Low risk: 3/3<br>Unclear: 0/3	High risk: 6/13<br>Low risk: 4/13<br>Unclear: 3/13	Unclear
**Panda 2022**[92]	High risk: 2/9<br>Low risk: 6/9<br>Unclear: 1/9	High risk: 0/3<br>Low risk: 3/3<br>Unclear: 0/3	High risk: 6/13<br>Low risk: 5/13<br>Unclear: 2/13	Unclear
**Pantazopoulos 2022**[93]	High risk: 2/9<br>Low risk: 6/9<br>Unclear: 1/9	High risk: 0/3<br>Low risk: 3/3<br>Unclear: 0/3	High risk: 6/13<br>Low risk: 4/13<br>Unclear: 3/13	Unclear
**Paranjape 2021**[94]	High risk: 2/9<br>Low risk: 6/9<br>Unclear: 1/9	High risk: 0/3<br>Low risk: 3/3<br>Unclear: 0/3	High risk: 6/13<br>Low risk: 4/13<br>Unclear: 3/13	Unclear

Author, year	Reporting	External validity	Confounding	Power
**Pham 2023**[95]	High risk: 0/9<br>Low risk: 7/9<br>Unclear: 2/9	High risk: 0/3<br>Low risk: 3/3<br>Unclear: 0/3	High risk: 5/13<br>Low risk: 7/13<br>Unclear: 1/13	Low risk
**Pivato 2022**[96]	High risk: 5/9<br>Low risk: 2/9<br>Unclear: 2/9	High risk: 1/3<br>Low risk: 2/3<br>Unclear: 0/3	High risk: 7/13<br>Low risk: 4/13<br>Unclear: 2/13	Unclear
**Platzer 2023**[97]	High risk: 3/9<br>Low risk: 4/9<br>Unclear: 2/9	High risk: 0/3<br>Low risk: 3/3<br>Unclear: 0/3	High risk: 5/13<br>Low risk: 5/13<br>Unclear: 3/13	Unclear
**Poliseno 2021**[98]	High risk: 3/9<br>Low risk: 4/9<br>Unclear: 2/9	High risk: 0/3<br>Low risk: 3/3<br>Unclear: 0/3	High risk: 6/13<br>Low risk: 4/13<br>Unclear: 3/13	Unclear
**Rai 2022**[99]	High risk: 2/9<br>Low risk: 5/9<br>Unclear: 2/9	High risk: 1/3<br>Low risk: 2/3<br>Unclear: 0/3	High risk: 6/13<br>Low risk: 5/13<br>Unclear: 2/13	Unclear
**Rawat 2022**[100]	High risk: 7/9<br>Low risk: 2/9<br>Unclear: 0/9	High risk: 1/3<br>Low risk: 0/3<br>Unclear: 2/3	High risk: 6/13<br>Low risk: 3/13<br>Unclear: 4/13	Unclear
**Razzaq 2022**[101]	High risk: 3/9<br>Low risk: 5/9<br>Unclear: 1/9	High risk: 0/3<br>Low risk: 3/3<br>Unclear: 0/3	High risk: 6/13<br>Low risk: 4/13<br>Unclear: 3/13	Unclear
**Read 2023**[102]	High risk: 7/9<br>Low risk: 2/9<br>Unclear: 0/9	High risk: 0/3<br>Low risk: 1/3<br>Unclear: 2/3	High risk: 6/13<br>Low risk: 3/13<br>Unclear: 4/13	Unclear
**Rivera 2023**[103]	High risk: 2/9<br>Low risk: 6/9<br>Unclear: 1/9	High risk: 0/3<br>Low risk: 3/3<br>Unclear: 0/3	High risk: 5/13<br>Low risk: 7/13<br>Unclear: 1/13	Unclear
**Russo 2021**[104]	High risk: 4/9<br>Low risk: 5/9<br>Unclear: 0/9	High risk: 0/3<br>Low risk: 3/3<br>Unclear: 0/3	High risk: 4/13<br>Low risk: 6/13<br>Unclear: 3/13	Unclear
**Russo 2022**[105]	High risk: 2/9<br>Low risk: 6/9<br>Unclear: 1/9	High risk: 1/3<br>Low risk: 0/3<br>Unclear: 2/3	High risk: 5/13<br>Low risk: 6/13<br>Unclear: 2/13	Unclear
**Said 2023**[106]	High risk: 3/9<br>Low risk: 5/9<br>Unclear: 1/9	High risk: 0/3<br>Low risk: 1/3<br>Unclear: 2/3	High risk: 6/13<br>Low risk: 4/13<br>Unclear: 3/13	Unclear
**Sattoju 2023**[107]	High risk: 3/9<br>Low risk: 5/9<br>Unclear: 1/9	High risk: 1/3<br>Low risk: 0/3<br>Unclear: 2/3	High risk: 6/13<br>Low risk: 4/13<br>Unclear: 3/13	Unclear
**Sellers 2023**[108]	High risk: 4/9<br>Low risk: 4/9<br>Unclear: 1/9	High risk: 0/3<br>Low risk: 1/3<br>Unclear: 2/3	High risk: 6/13<br>Low risk: 3/13<br>Unclear: 4/13	Unclear
**Senthiappan 2023**[109]	High risk: 4/9<br>Low risk: 4/9<br>Unclear: 1/9	High risk: 0/3<br>Low risk: 1/3<br>Unclear: 2/3	High risk: 6/13<br>Low risk: 3/13<br>Unclear: 4/13	Unclear
**So 2021**[110]	High risk: 2/9<br>Low risk: 5/9<br>Unclear: 2/9	High risk: 0/3<br>Low risk: 2/3<br>Unclear: 1/3	High risk: 5/13<br>Low risk: 5/13<br>Unclear: 3/13	Unclear
**Spagnuolo 2022**[111]	High risk: 2/9<br>Low risk: 5/9<br>Unclear: 2/9	High risk: 0/3<br>Low risk: 3/3<br>Unclear: 0/3	High risk: 6/13<br>Low risk: 5/13<br>Unclear: 2/13	Unclear
**Ughi 2023**[112]	High risk: 0/9<br>Low risk: 8/9<br>Unclear: 1/9	High risk: 0/3<br>Low risk: 1/3<br>Unclear: 2/3	High risk: 5/13<br>Low risk: 7/13<br>Unclear: 1/13	Low risk

Author, year	Reporting	External validity	Confounding	Power
**Umeh 2023**[113]	High risk: 4/9<br>Low risk: 4/9<br>Unclear: 1/9	High risk: 0/3<br>Low risk: 3/3<br>Unclear: 0/3	High risk: 6/13<br>Low risk: 4/13<br>Unclear: 3/13	Unclear
**Viray 2023**[114]	High risk: 5/9<br>Low risk: 1/9<br>Unclear: 3/9	High risk: 1/3<br>Low risk: 0/3<br>Unclear: 2/3	High risk: 6/13<br>Low risk: 3/13<br>Unclear: 4/13	Unclear
**Williams 2023**[115]	High risk: 4/9<br>Low risk: 4/9<br>Unclear: 1/9	High risk: 0/3<br>Low risk: 3/3<br>Unclear: 0/3	High risk: 6/13<br>Low risk: 4/13<br>Unclear: 3/13	Unclear
**Wong 2022a**[116]	High risk: 1/9<br>Low risk: 7/9<br>Unclear: 1/9	High risk: 0/3<br>Low risk: 3/3<br>Unclear: 0/3	High risk: 6/13<br>Low risk: 7/13<br>Unclear: 0/13	Unclear
**Wong 2022b**[117]	High risk: 3/9<br>Low risk: 5/9<br>Unclear: 1/9	High risk: 0/3<br>Low risk: 3/3<br>Unclear: 0/3	High risk: 5/13<br>Low risk: 6/13<br>Unclear: 2/13	Unclear
**Wu 2022**[118]	High risk: 2/9<br>Low risk: 6/9<br>Unclear: 1/9	High risk: 0/3<br>Low risk: 1/3<br>Unclear: 2/3	High risk: 5/13<br>Low risk: 6/13<br>Unclear: 2/13	Unclear

Supplemental Table S8. **Remdesivir efficacy and effectiveness on 28-30 day mortality in hospitalized adults with COVID-19 by oxygen support level**

Study	Primary efficacy outcome	Study period	Sample size, N	Measure of estimate	Effect estimates† RCT	Effect estimates† RWD
			Overall			
**Bechman, 2022**[37]	Mortality at 28 days	Mar 20 - Feb 21	3,949	aHR		0.83 (0.63-1.09)
**Beigel, 2020**[9], **ACTT-1**	Time to recovery	Feb 20 - May 20	1,062	HR	0.73 (0.52-1.03)	
**Benfield 2021**[39ʃʃ]	Survival status at 30 days; mechanical ventilation	Feb 20 - Dec 20	2,747	OR		**0.47 (0.38-0.57)**
**Caffrey, 2023**[47ʃ]	Time to inpatient mortality	May 20 - Nov 21	16,416	HR		**0.80 (0.69-0.92)**
**Chokkalingam, 2022**[51ʃ]	Time to inpatient mortality	May 20 - May 21	49,712	HR		**0.83 (0.79-0.87)**
**De Vito, 2022**[54ʃ]	Mortality at 28 days	Aug 20 - Oct 21	1,080	HR		**0.16 (0.10-0.26)**
**Diaz, 2022**[58ʃ]	Overall survival	Feb 20 - May 20	1,138	OR		**0.56 (0.32-0.97)**
**Dobrowolska 2023**[59ʃ]	Need for oxygen therapy; Need for mechanical ventilation; Mortality at 28 days	May 21 - Nov 22	1,170	OR		**0.42 (0.29-0.60)**
	LOS, 30-day readmission; Post-discharge 30-day mortality	Dec 21 - Apr 22	652	OR		**0.56 (0.35-0.92)**
**Finn, 2022**[63]	Time to inpatient mortality	Apr 20 - Dec 20	2,111	HR		**0.65 (0.49-0.85)**
**Garibaldi, 2022**[64ʃ]	Time to clinical improvement	Feb 20 - Feb 21	18,328	aHR		1.02 (0.97-1.08)
**Leding, 2023**[75ʃʃ]	Use of IMV; Mortality at 30 days	Feb 20 - Apr 21	3,826	OR		**0.47 (0.39-0.56)**

Study	Primary efficacy outcome	Study period	Sample size, N	Measure of estimate	Effect estimates†	
					RCT	RWD
**Marx, 2022[82]**	Time to clinical improvement	Jul 20 - Jun 21	88	OR		**0.20 (0.04-0.68)**
**Mozaffari, 2022[88]ʃ**	Inpatient mortality at 14 days and 28 days	Aug 20 - Nov 20	57,710	HR		**0.76 (0.69-0.83)**
**Mozaffari, 2023[89]**	Mortality at 14 days and 28 days	Dec 21 - Apr 22	24,154	HR		**0.76 (0.67-0.85)**
**Olender, 2021[119], SIMPLE-Severe**	Clinical recovery at 14 days; Mortality at 28 days	Feb 20 - May 20	1,747	aOR	**0.67 (0.47-0.95)**	
**Henao-Restrepo, 2022[121], SOLIDARITY**	Mortality at 28 days	Mar 20 - Jan 21	1,730	RR	0.91 (0.82-1.02)	
**Wang, 2020[24]ʃ**	Time to clinical improvement	Feb 20 - Mar 20	227	RD	-3.6 (-16.2-8.9)	
		No supplemental oxygen at admission				
**ACTT-1, Beigel, 2020[9]**, mild-moderate	Time to recovery	Feb 20 - May 20	105	HR	0.60 (0.10-1.56)	
**ACTT-1, Beigel, 2020[9]**	Time to recovery	Feb 20 - May 20	138	HR	0.82 (0.17-1.60)	
**Caffrey, 2023[47]ʃ**	Time to inpatient mortality	May 20 - Nov 21	2,276	HR		1.09 (0.75-1.58)
**Chokkalingam, 2022[51]ʃ**	Time to inpatient mortality	May 20 - May 21	31,418	HR		**0.88 (0.82-0.94)**
**Diaz, 2022[58]ʃ**	Overall survival	Feb 20 - May 20	202	HR		1.10 (0.10-1.76)
**Finn, 2022[63]**	Time to inpatient mortality	Apr 20 - Dec 20	806	HR		0.45 (0.12-1.65)
**Garibaldi, 2022[64]ʃ**	Time to clinical improvement	Feb 20 - Feb 21	5,332	HR		1.08 (0.92-1.27)
**Mozaffari, 2023[89]**	Mortality at 14 days and 28 days	Dec 21 - Apr 22	24,154	HR		**0.76 (0.67-0.85)**

Study	Primary efficacy outcome	Study period	Sample size, N	Measure of estimate	Effect estimates†	
					RCT	RWD
**Mozaffari, 2023**[89]	Mortality at 14 days and 28 days	May 21 - Nov 21	45,598	HR		**0.85 (0.75-0.96)**
**Mozaffari, 2023**[89]	Mortality at 14 days and 28 days	Dec 20 - Apr 21	46,624	HR		**0.82 (0.71-0.95)**
**Mozaffari, 2022**[88]ʃ	Inpatient mortality at 14 days and 28 days	Aug 20 - Nov 20	15,940	HR		**0.80 (0.68-0.94)**
**ACTT-1, Paules, 2022**[120]	Risk profile for progression to IMV or death	Feb 20 - May 20	138	HR	0.56 (0.16-1.97)	
**Leding, 2023**[75]ʃʃ	Use of IMV; Mortality at 30 days	Feb 20 - Apr 21	1,248	OR		0.95 (0.69-1.31)
**Olender, 2021**[119]**, SIMPLE-Severe**	Clinical recovery at 14 days; Mortality at 28 days	Feb 20 - May 20	249	OR		1.13 (0.41-2.00)
**Henao-Restrepo, 2022**[121]**, SOLIDARITY**	Mortality at 28 days	Mar 20 - Jan 21	1,730	RR	0.76 (0.46-1.28)	
**Breskin, 2023**[43]**,** RDV vs no RDVʃ	Mortality at 30 days; incidence of IMV/ECMO	May 20 - Dec 21	71,068**	RD	-0.21 (-0.87-0.44)	
**Breskin, 2023**[43]**,** RDV vs no early RDVʃ	Mortality at 30 days; incidence of IMV/ECMO	May 20 - Dec 21		RD	-0.07 (-0.63-0.50)	
			Low flow oxygen			
**ACTT-1, Beigel, 2020**[9]	Time to recovery	Feb 20 - May 20	435	HR	**0.30 (0.14-0.64)**	
**Caffrey, 2023**[47]ʃ	Time to inpatient mortality	May 20 - Nov 21	12,457	HR		**0.71 (0.60-0.84)**
**Chokkalingam, 2022**[51]ʃ	Time to inpatient mortality	May 20 - May 21	11,046	HR		**0.81 (0.73-0.90)**
**Diaz, 2022**[58]ʃ	Overall survival	Feb 20 - May 20	827	HR		0.63 (0.39-1.00)

Study	Primary efficacy outcome	Study period	Sample size, N	Measure of estimate	Effect estimates†	
					RCT	RWD
**ACTT-1, Fintzi, 2022[122]**	Clinical outcomes	Feb 20 - May 20	435	HR	**0.36 (0.16-0.77)**	
**Garibaldi, 2022[64ʃ]**	Time to clinical improvement	Feb 20 - Feb 21	20,966	HR		**0.85 (0.77-0.92)**
**Marx, 2022[82],** RDV vs SOC	Time to clinical improvement	Jul 20 - Jun 21	67	HR		0.23 (0.05-1.09)
**Mozaffari, 2023[89]**	Mortality at 14 days and 28 days	Dec 21 - Apr 22	24,616	HR		**0.74 (0.66-0.82)**
**Mozaffari, 2023[89]**	Mortality at 14 days and 28 days	May 21 - Nov 21	57,638	HR		**0.81 (0.73-0.90)**
**Mozaffari, 2023[89]**	Mortality at 14 days and 28 days	Dec 20 - Apr 21	52,910	HR		**0.79 (0.70-0.90)**
**Mozaffari, 2022[88ʃ]**	Inpatient mortality at 14 days and 28 days	Aug 20 - Nov 20	27,616	HR		**0.76 (0.68-0.86)**
**Henao-Restrepo, 2022[121], SOLIDARITY**	Mortality at 28 days	Mar 20 - Jan 21	1,730	RR	0.87 (0.76-0.99)	
**Leding, 2023[75ʃʃ]**	Use of IMV; Mortality at 30 days	Feb 20 - Apr 21	2,046	OR		0.43 (0.13-1.44)
**Marx, 2022[82],** RDV vs SOC	Time to clinical improvement	Jul 20 - Jun 21	67	OR		**0.21 (0.03-0.89)**
**SIMPLE-Severe[µ], Olender, 2021[119]**	Clinical recovery at 14 days; Mortality at 28 days	Feb 20 - May 20	1,013	OR		**0.29 (0.14-0.58)**
**Breskin, 2023[43],** RDV vs no RDV[ʃ]	Mortality at 30 days; incidence of IMV/ECMO	May 20 - Dec 21	180,248**	RD	**-4.20 (-4.70-(-3.60))**	
**Breskin, 2023[43],** RDV vs no early RDV[ʃ]	Mortality at 30 days; incidence of IMV/ECMO	May 20 - Dec 21		RD	**-3.50 (-3.90-(-3.00))**	

Study	Primary efficacy outcome	Study period	Sample size, N	Measure of estimate	Effect estimates[†]	
					RCT	RWD
**Henao-Restrepo, 2022[121], SOLIDARITY**	Mortality at 28 days	Mar 20 - Jan 21	1,730	RR	0.87 (0.76-0.99)	
High flow oxygen						
**ACTT-1, Beigel, 2020[9]**	Time to recovery	Feb 20 - May 20	193	HR	1.02 (0.54-1.91)	
**Caffrey, 2023[47ʃ]**	Time to inpatient mortality	May 20 - Nov 21	1,319	HR		1.36 (0.81-2.28)
**Chokkalingam, 2022[51ʃ]**	Time to inpatient mortality	May 20 - May 21	5,292	HR		**0.77 (0.71-0.85)**
**Diaz, 2022[58ʃ]**	Overall survival	Feb 20 - May 20	77	HR		0.72 (0.19-2.70)
**Garibaldi, 2022[64ʃ]**	Time to clinical improvement	Feb 20 - Feb 21	7,615	HR		1.10 (1.01-1.20)
**Marx, 2022[82]**	Time to clinical improvement	Jul 20 - Jun 21	147	OR		0.73 (0.36-1.49)
**Mozaffari, 2023[89]**	Mortality at 14 days and 28 days	Dec 21 - Apr 22	15,062	HR		**0.84 (0.76-0.93)**
**Mozaffari, 2023[89]**	Mortality at 14 days and 28 days	May 21 - Nov 21	30,332	HR		**0.89 (0.82-0.97)**
**Mozaffari, 2023[89]**	Mortality at 14 days and 28 days	Dec 20 - Apr 21	24,320	HR		**0.88 (0.80-0.98)**
**Mozaffari, 2022[88ʃ]**	Inpatient mortality at 14 days and 28 days	Aug 20 - Nov 20	11,562	HR		0.97 (0.84-1.11)
**Marx 2022[82]**	Time to clinical improvement	Jul 20 - Jun 21	147	HR		0.75 (0.41-1.37)
**Leding, 2023[75ʃʃ]**	Use of IMV; Mortality at 30 days	Feb 20 - Apr 21	297	OR		**0.23 (0.09-0.55)**
**Henao-Restrepo, 2022[121], SOLIDARITY**	Mortality at 28 days	Mar 20 - Jan 21	1,730	RR	0.87 (0.76-0.99)	

Study	Primary efficacy outcome	Study period	Sample size, N	Measure of estimate	Effect estimates[†]	
					RCT	RWD
**SIMPLE-Severe[µ], Olender, 2021[119]**	Clinical recovery at 14 days; Mortality at 28 days	Feb 20 - May 20	445	OR		1.01 (0.62-1.65)
**Breskin, 2023[43],** RDV vs no RDV[ʃ]	Mortality at 30 days; incidence of IMV/ECMO	May 20 - Dec 21	14,912**	RD	**-8.30 (-12.00-(-5.00))**	
**Breskin, 2023[43],** RDV vs no early RDV[ʃ]	Mortality at 30 days; incidence of IMV/ECMO	May 20 - Dec 21		RD	**-7.40 (-10.00-(-4.90))**	
Invasive mechanical ventilation and/or extracorporeal membrane oxygenation						
**ACTT-1, Beigel, 2020[9]**	Time to recovery	Feb 20 - May 20	285	HR	1.13 (0.67-1.89)	
**Caffrey, 2023[47ʃ]**	Time to inpatient mortality	May 20 - Nov 21	364	HR		0.88 (0.41-1.90)
**Chokkalingam, 2022[51ʃ]**	Time to inpatient mortality	May 20 - May 21	1,456	HR		**0.83 (0.73-0.95)**
**Garibaldi, 2022[64ʃ]**	Time to clinical improvement	Feb 20 - Feb 21	2,743	HR		**1.17 (1.04-1.32)**
**Lapadula, 2020[74]**	Time to mortality; Time to hospital discharge	Mar 20 - Apr 20	113	HR		0.73 (0.26-2.09)
**Mozaffari, 2023[89]**	Mortality at 14 days and 28 days	Dec 21 - Apr 22	2,020	HR		**0.71 (0.61-0.83)**
**Mozaffari, 2023[89]**	Mortality at 14 days and 28 days	May 21 - Nov 21	3,530	HR		**0.81 (0.69-0.95)**
**Mozaffari, 2023[89]**	Mortality at 14 days and 28 days	Dec 20 - Apr 21	2,778	HR		**0.69 (0.58-0.82)**
**Mozaffari, 2022[88ʃ]**	Inpatient mortality at 14 days and 28 days	Aug 20 - Nov 20	2,592	HR		**0.81 (0.69-0.94)**
**Henao-Restrepo, 2022[121], SOLIDARITY**	Mortality at 28 days	Mar 20 - Jan 21	706	RR	1.13 (0·89–1·42)	

Study	Primary efficacy outcome	Study period	Sample size, N	Measure of estimate	Effect estimates†	
					RCT	RWD
**Leding, 2023[75]∫∫**	Use of IMV; mortality at 30 days	Feb 20 - Apr 21	51	OR		0.43 (0.13-1.44)
**SIMPLE-Severe[μ], Olender, 2021[119]**	Clinical recovery at 14 days; mortality at 28 days	Feb 20 - May 20	60	OR		0.97 (0.27-3.44)
**Breskin, 2023[43]**, RDV vs no RDV[∫]	Mortality at 30 days; incidence of IMV/ECMO	May 20 - Dec 21	8,091**	RD	**-6.20 (-9.60-(-2.80))**	
**Breskin, 2023[43]**, RDV vs no early RDV[∫]	Mortality at 30 days; incidence of IMV/ECMO	May 20 - Dec 21		RD	**-5.80 (-8.80-(-2.80))**	

†Statistically significant results are bolded.
* Any oxygenation status at entry.
** The study did not report number of individuals for each arm among different oxygen therapy subgroups.
∫ Corticosteroids were used in both remdesivir and no remdesivir groups.
∫∫ Corticosteroids were used in all patients receiving remdesivir.
μ Excluding mechanically ventilated patients.
Notes: The Solidarity trial results were not differentiated by low-flow and high-flow for patients receiving oxygen support at admission.
CS, corticosteroids; HFNC, high flow nasal cannula; HR, hazard ratio; IMV, invasive mechanical ventilation; LOS, length of hospital stay; OR, odds ratio; RCT, randomized controlled trial; RDV, remdesivir; RD, risk difference; RR, rate ratio; RWD, real world data.

Supplemental Table S9. Characteristics of studies reporting remdesivir efficacy and effectiveness on time to clinical improvement in adults with COVID-19

Author, Year, Study Name	Study period	Location, Setting	Study design	Sample size, N	Proportion of patients not requiring supplemental oxygen at baseline	Duration of remdesivir treatment	Clinical improvement criteria	Primary efficacy outcome
**Ader, 2021[14], DisCoVeRy**ʃ	Mar 20 - Jan 21	Austria; Belgium; France; Luxembourg; Portugal; Multicentric	RCT	832	NR	5 or 10 days	NEWS ≤2 or hospital discharge within 29 days.	Clinical status at 15 days (WHO ordinal scale)
**Beigel, 2020[9], ACTT-1**	Feb 20 - May 20	Denmark; Germany; Greece; Japan; Korea; Mexico; Singapore; Spain; United Kingdom; United States; Multicentric	RCT	1,062	12% in treated 14% in untreated	up to 10 days	The first day a patient met the criteria for category 1, 2, or 3 on OS.	Time to recovery
**Garibaldi, 2022[64]**ʃ	Feb 20 - Feb 21	United States; Multicentric	RW, retrospective	36,656	15.6% in treated 13.5% in untreated	5 days	A 2-point decrease in the 8-point WHO SS or discharged alive without worsening of the WHO SS.	Time to clinical improvement
**Viray, 2023[114]**	Sep 20 - Sep 21	Philippines; Sigle center	RW, retrospective	318	NR	NR	NR	Mortality; Time to clinical improvement
**Wang, 2020[24]**ʃ	Feb 20 - Mar 20	China; Multicentric	RCT	236	0% in treated 4% in untreated	10 days	Decline of two categories on the modified six-	Time to clinical improvement

						category OS of clinical status, or hospital discharge.

ʃ Corticosteroids were used in both remdesivir and no remdesivir groups.
NEWS, National Early Warning Score; NR, not reported; OS, ordinal score; RCT, randomized controlled trial; RW, real world ; WHO SS, World Health Organization's severity score.

Supplemental Table S10. **Remdesivir efficacy and effectiveness on time to clinical improvement in hospitalized adults with COVID-19**

Study	Primary efficacy outcome	Study period	Sample size, N	Measure of estimate	Effect estimates†	
					RCT	RWD
			Overall population			
**Ader, 2021[14], DisCoVeRy**ʃ	Clinical status at 15 days (WHO ordinal scale)	Mar 20 - Jan 21	832	HR	1.03 (0.88-1.21)	
**Beigel, 2020[9], ACTT-1**	Time to recovery	Feb 20 - May 20	1,062	RR	**1.27 (1.1-1.46)**	
**Garibaldi, 2022[64]**ʃ	Time to clinical improvement	Feb 20 - Feb 21	1,856	aHR		**1.19 (1.16-1.22)**
**Viray, 2023[114]**	Mortality; Time to clinical improvement; Time to clinical recovery	Sep 20 - Sep 21	318	MD		**p=0.00**
**Wang, 2020[24]**ʃ	Time to clinical improvement	Feb 20 - Mar 20	236	HR	1.23 (0.87-1.75)	

ʃ Corticosteroids were used in both remdesivir and no remdesivir groups.
aHR, adjusted hazard ratio; HR, hazard ratio; MD, mean difference; RR, relative risk; RCT, randomized controlled trial; RWD, real world data.

Supplemental Table S11. **Characteristics of studies reporting remdesivir efficacy and effectiveness on time to clinical recovery in adults with COVID-19**

Author, Year,	Study period	Location, Setting	Study design	Sample size, N	Proportion of patients not requiring	Duration of remdesivir treatment	Clinical recovery criteria	Primary efficacy outcome

Study Name					supplemental oxygen at baseline			
**Beigel, 2020[9], ACTT-1**	Feb 20 - May 20	Denmark; Germany; Greece; Japan; Korea; Mexico; Singapore; Spain; United Kingdom; United States; Multicentric	RCT	1,062	12% in treated 14% in untreated	up to 10 days	The first day a patient met the criteria for category 1, 2, or 3 on OS.	Time to recovery
**Sellers, 2023[108]**	Jul 20 - Sep 21	United States; Single center	RW, retrospective	300	3.5% in treated 16% in untreated	NR	Discharge from the hospital or no longer requiring supplemental oxygen.	Time to recovery
**Viray, 2023[114]**	Sep 20 - Sep 21	Philippines; Sigle center	RW, retrospective	318	NR	NR	NR	Mortality; Time to clinical improvement; Time to clinical recovery

NR, not reported; OS, ordinal score; RCT, randomized controlled trial; RW, real world.

Supplemental Table S12. Remdesivir efficacy and effectiveness on time to clinical recovery in hospitalized adults with COVID-19

Study	Primary efficacy outcome	Study period	Sample size, N	Measure of estimate	Effect estimates†	
					RCT	RWD
			Overall population			
**Beigel, 2020[9], ACTT-1**	Time to recovery	Feb 20 - May 20	1,062	RR	**1.29 (1.12-1.49)**	
**Sellers, 2023[108]**	Time to recovery	Jul 20 - Sep 21	300	MD		**p=0.035**

Study	Primary efficacy outcome	Study period	Sample size, N	Measure of estimate	Effect estimates†	
					RCT	RWD
**Viray, 2023[114]**	Mortality; Time to clinical improvement; Time to clinical recovery	Sep 20 - Sep 21	318	MD		**p=0.00**

HR, hazard ratio; MD, mean difference; RCT, randomized controlled trial; RWD, real world data.

Supplemental Table S13. **Characteristics of studies reporting remdesivir efficacy and effectiveness on risk of rehospitalization in adults with COVID-19**

Author, Year, Study Name	Study period	Location, Setting	Study design	Sample size, N	Proportion of patients not requiring supplemental oxygen at baseline	Duration of remdesivir treatment	Primary efficacy outcome
**Beigel, 2020[9], ACTT-1**	Feb 20 - May 20	Denmark; Germany; Greece; Japan; Korea; Mexico; Singapore; Spain; United Kingdom; United States; Multicentric	RCT	1,062	12% in treated<br>14% in untreated	up to 10 days	Time to recovery
**Caffrey, 2023[47∫]**	May 20 - Nov 21	United States; Multicentric	RW, retrospective	18,874	17.7% in treated<br>15.9% in untreated	NR	Time to inpatient mortality
**Finn, 2022[63]**	Apr 20 - Dec 20	United States; Multicentric	RW, prospective	2,230	NR	NR	Time to inpatient mortality
**Mozaffari, 2023[90]**	May 20 - Apr 22	United States; Multicentric	RW, retrospective	440,601	30% in treated<br>52% in untreated	NR	Hospital readmission at 30 days

∫ Corticosteroids were used in both remdesivir and no remdesivir groups.
NR, not reported; RCT, randomized controlled trial; RW, real world.

Supplemental Table S14. **Remdesivir efficacy and effectiveness on risk of rehospitalization among hospitalized adults with COVID-19**

Study	Primary efficacy outcome	Study period	Sample size, N	Measure of estimate	Effect estimates†	
					RCT	RWD
			Overall population			

Study	Primary efficacy outcome	Study period	Sample size, N	Measure of estimate	Effect estimates†	
					RCT	RWD
**Beigel, 2020[9], ACTT-1**	Time to recovery	Feb 20 - May 20	1,062	Difference	2.00 (0.00-4.00)	
**Caffrey, 2023[47ʃ]**	Time to inpatient mortality	May 20 - Nov 21	16,416	HR		**0.74 (0.59-0.93)**
**Finn, 2022[63]**	Time to inpatient mortality	Apr 20 - Dec 20	2,111	RR		**0.31 (0.13-0.75)**
**Mozaffari, 2023[90]**	Hospital readmission at 30 days	May 20 - Apr 22	440,601	OR		**0.70 (0.67-0.73)**

ʃ Corticosteroids were used in both remdesivir and no remdesivir groups.
HR, hazard ratio; RCT, randomized controlled trial; RR, rate ratio; RWD, real world data.

Supplemental Table S15. Complete list of included studies

Author, year	Title	Journal	Volume	Issue	Page	Study type
Acharya 2022	Study of Clinical Outcome and Healthcare Modalities of COVID-19 Patients Treated With Remdesivir at a Tertiary Care Teaching Hospital	Cureus	14	1	e21535	RW
Ader 2021	An open-label randomized controlled trial of the effect of lopinavir/ritonavir, lopinavir/ritonavir plus IFN-β-1a and hydroxychloroquine in hospitalized patients with COVID-19	Clinical Microbiology and Infection	27	12	1826–1837	RCT
Ahmed 2022	Remdesivir Treatment for COVID-19 in Hospitalized Children: CARAVAN Interim Results	Topics in Antiviral Medicine	30	Suppl 1	296-297	RW
Aiello 2023	Non-supervised artificial intelligence clustering algorithm identifies patients hospitalised with COVID-19 in whom remdesivir decreases mortality	ECCMID 2023	-	-	-	RW
Aksak-Was 2022	Remdesivir Reduces Mortality in Hemato-Oncology Patients with COVID-19	Journal of Inflammation Research	15	-	4907-4920	RW
Alexander 2023	Evaluation of Remdesivir to the outcomes of hospitalized patients with COVID-19 infection in a tertiary-care hospital in southern India	Journal of Infection in Developing Countries	17	3	304–310	RW
Ali 2022	Remdesivir for the treatment of patients in hospital with COVID-19 in Canada: a randomized controlled trial	CMAJ	-	-	-	RCT
Alshamrani 2023	Comprehensive evaluation of six interventions for hospitalized patients with COVID-19: A propensity score matching study	Saudi Pharmaceutical Journal	31	4	517–525	RW
Arribas López 2023	Remdesivir associated with decreased mortality in hospitalised COVID-19 patients: a real-world evidence study using natural language processing	ECCMID 2023	-	-	-	RW
Attena 2023	Remdesivir treatment and clinical outcome in non-severe hospitalized COVID-19 patients: a	European Journal of Clinical Pharmacology	79	-	967–974	RW

Author, year	Title	Journal	Volume	Issue	Page	Study type
	propensity score matching multicenter Italian hospital experience					
Ayodele 2021	Real-world treatment patterns and clinical outcomes for inpatients with COVID-19 in the US from September 2020 to February 2021	PLoS ONE [Electronic Resource]	16	12	e0261707	RW
Bansal 2021	A shorter symptom onset to remdesivir treatment (SORT) interval is associated with a lower mortality in moderate to severe COVID-19: A real-world analysis	American Journal of Respiratory and Critical Care Medicine. Conference: American Thoracic Society International Conference, ATS	203	9	-	RW
Bárczi 2023	Formerly hospitalized patients treated with remdesivir presented less respiratory post-COVID symptoms	ERS 2023	-	-	-	RW
Barratt-Due 2021	Evaluation of the effects of remdesivir and hydroxychloroquine on viral clearance in covid-19: A randomized trial	Annals of Internal Medicine	174	9	1261–1269	RCT
Basoulis 2023	Safety and effectiveness of three-day remdesivir regimen to prevent progression to severe COVID-19: a single-centre, real-world study	ECCMID 2023	-	-	-	RW
Bavaro 2023	Efficacy of Remdesivir and Neutralizing Monoclonal Antibodies in Monotherapy or Combination Therapy in Reducing the Risk of Disease Progression in Elderly or Immunocompromised Hosts Hospitalized for COVID-19: A Single Center Retrospective Study	Viruses	15	5	-	RW
Bechman 2022	Inpatient COVID-19 mortality has reduced over time: Results from an observational cohort	PloS ONE	17	44927	-	RW
Beigel 2020	Remdesivir for the Treatment of Covid-19 - Final Report	New England Journal of Medicine	383	19	1813–1826	RCT
Behboodikhah 2022	Evaluation of the Costs and Outcomes of COVID-19 Therapeutic Regimens in Hospitalized Patients in Shiraz	Iranian Journal of Science & Technology Transaction a Science	46	5	1339-1347	RW

Author, year	Title	Journal	Volume	Issue	Page	Study type
Benfield 2021	Improved Survival Among Hospitalized Patients With Coronavirus Disease 2019 (COVID-19) Treated With Remdesivir and Dexamethasone. A Nationwide Population-Based Cohort Study	Clinical Infectious Diseases	73	11	2031–2036	RW
Bernal 2023	Remdesivir plus dexamethasone is associated to improvement in the clinical outcome of COVID-19 hospitalized patients regardless of their vaccination status	Medicina Clinica	161	4	139–146	RW
Bistrovic 2022	Bradycardia during remdesivir treatment might be associated with improved survival in patients with COVID-19: a retrospective cohort study on 473 patients from a tertiary centre	Postgraduate medical journal	98	1161	501–502	RW
Boglione 2022	Remdesivir treatment in hospitalized patients affected by COVID-19 pneumonia: A case-control study	Journal of Medical Virology	94	8	3653–3660	RW
Breskin 2023	Effectiveness of Remdesivir Treatment Protocols Among Patients Hospitalized with COVID-19: A Target Trial Emulation	Epidemiology	34	3	365–375	RW
Burhan 2023	Evaluation of safety and effectiveness of remdesivir in treating COVID-19 patients after emergency use authorization study	Frontiers in Pharmacology	14	-	-	RW
Butt 2023	Risk of Death in Remdesivir Treated and Untreated Patients with Covid-19 Infection	Topics in Antiviral Medicine	31	2	-	RW
Caffrey 2021	Utilization of Remdesivir for COVID-19 in the National Veterans Affairs Healthcare System	Open Forum Infectious Diseases	8	Suppl 1	S380	RW
Caffrey 2023	Real-World Safety and Effectiveness of Remdesivir and Corticosteroids in Hospitalized Patients with COVID-19	COVID	3	2	198-217	RW
Chaudhary 2023	Study of Efficacy of Injection Remdesivir in Patients of COVID-19	Journal of the Association of Physicians of India	71	4	52–56	RW
Chavalertsakul 2023	Remdesivir versus favipiravir in hospitalized patients with moderate COVID-19	ERS 2023	-	-	-	RW

Author, year	Title	Journal	Volume	Issue	Page	Study type
	pneumonia: A propensity score-matched retrospective cohort study.					
Chokkalingam 2022	Association of Remdesivir Treatment With Mortality Among Hospitalized Adults With COVID-19 in the United States	JAMA network open	5	12	e2244505	RW
Cilloniz 2023	Remdesivir and survival outcomes in critically ill patients with COVID-19: A multicentre observational cohort study	Journal of Infection	86	3	256–308	RW
Das 2022	Mortality comparison in patients receiving either Remdesivir or Remdesivir plus Baricitinib combination in case of moderate to severe COVID-19 Pneumonia: A retrospective study	The Journal of the Association of Physicians of India	70	4	11–12	RW
De Vito 2022	Efficacy of remdesivir in reducing mortality in people hospitalized for SARS-CoV-2 infection: a real-life experience	ECCMID 2022	-	-	-	RW
De Vito 2023	Safety and efficacy of remdesivir three-day course in SARS-CoV-2 infected patients: a real-life experience	ECCMID 2023	-	-	-	RW
Delgado 2023	Investigational medications in 9,638 hospitalized patients with severe COVID-19: lessons from the "fail-and-learn" strategy during the first two waves of the pandemic in 2020	Patient Safety in Surgery [Electronic Resource]	17	-	7	RW
Devgun 2023	Identification of Bradycardia Following Remdesivir Administration Through the US Food and Drug Administration American College of Medical Toxicology COVID-19 Toxic Pharmacovigilance Project	JAMA network open	6	2	e2255815	RW
Diaz 2022	Remdesivir and Mortality in Patients With Coronavirus Disease 2019	Clinical infectious diseases	74	10	1812-1820	RW
Dobrowolska 2023	Retrospective Analysis of the Effectiveness of Remdesivir in COVID-19 Treatment during Periods Dominated by Delta and Omicron SARS-CoV-2 Variants in Clinical Settings	Journal of Clinical Medicine	12	12	6	RW

Author, year	Title	Journal	Volume	Issue	Page	Study type
Elshaboury 2022	Short vs. Long Symptom Duration Prior to Remdesivir for Hospitalized Patients with COVID-19	Open Forum Infectious Diseases	9	Suppl 2	S495–S496	RW
Estill 2023	Treatment effect of remdesivir on the mortality of hospitalised COVID-19 patients in Switzerland across different patient groups: a tree-based model analysis	Swiss Medical Weekly	153	8	40095	RW
Falcone 2022	Early Use of Remdesivir and Risk of Disease Progression in Hospitalized Patients With Mild to Moderate COVID-19	Clinical Therapeutics	44	3	364–373	RW
Finn 2022	Association of Treatment with Remdesivir and 30-day Hospital Readmissions in Patients Hospitalized with COVID-19	American Journal of the Medical Sciences	363	5	403-410	RW
Garibaldi 2022	Real-World Effectiveness of Remdesivir in Adults Hospitalized With Coronavirus Disease 2019 (COVID-19): A Retrospective, Multicenter Comparative Effectiveness Study	Clinical Infectious Diseases	75	1	e516-e524	RW
Goldman 2020	Remdesivir for 5 or 10 days in patients with severe covid-19	New England Journal of Medicine	383	19	1827–1837	RCT
Gragera Gomez 2022	Experience of Baricitinib-Remdesivir Use in Patients with Sars-Cov-2 Infection	European Journal of Hospital Pharmacy	29	Suppl 1	A157	RW
Grundmann 2023	Fewer COVID-19 Neurological Complications with Dexamethasone and Remdesivir	Annals of Neurology	93	1	88–102	RW
Gunasekaran 2021	Does Remdesivir Impact the Clinical Outcome of Patients with COVID 19 Infection?	Open Forum Infectious Diseases	8	Suppl 1	S371-S372	RW
Henao-Restrepo 2022	Remdesivir and three other drugs for hospitalised patients with COVID-19: final results of the WHO Solidarity randomised trial and updated meta-analyses	The Lancet	399	10339	1941-1953	RCT
Hormati 2023	Comparison of Efficacy of Remdesivir with Supportive Care Alone in the Treatment of Critically Sick Adult and Child COVID-19 Patients: a Randomized Clinical Trial	Anti-Infective Agents	21	1	82–88	RCT
Jeyapalina 2022	Effectiveness of Remdesivir as Treatment for COVID-19 Positive US Veterans	Open Forum Infectious Diseases	9	Suppl 2	S480-S481	RW

Author, year	Title	Journal	Volume	Issue	Page	Study type
Jittamala 2023	Clinical Antiviral Efficacy of Remdesivir in Coronavirus Disease 2019: an Open-Label, Randomized Controlled Adaptive Platform Trial (PLATCOV)	Journal of Infectious Diseases	228	10	1318–1325	RCT
Kalil 2021a	Baricitinib plus Remdesivir for Hospitalized Adults with Covid-19	New England Journal of Medicine	384	9	795–807	RCT
Kalil 2021b	Efficacy of interferon beta-1a plus remdesivir compared with remdesivir alone in hospitalized adults with COVID-19: a double-bind, randomized, placebo-controlled, phase 3 trial	The Lancet Respiratory Medicine	9	12	1365–1376	RCT
Karolyi 2022	Early administration of remdesivir may reduce mortality in hospitalised COVID-19 patients: a propensity-score matched analysis	ECCMID 2022	-	-	-	RW
Koh 2023	Real-world effectiveness of sotrovimab and remdesivir for early treatment of high-risk hospitalized COVID-19 patients: A propensity score adjusted retrospective cohort study	Journal of Medical Virology	95	2	e28460	RW
Kubiliute 2023	Systemic dexamethasone plus remdesivir and dexamethasone alone effect on in-hospital mortality among COVID-19 patients hospitalised in the university hospital, Lithuania	ECCMID 2023	-	-	-	RW
Lakhanpal 2022	Reduction in the Rate of Mortality of Moderate to Severe COVID 19 Infected Patients with the use of Remdesivir - A Tertiary Care Hospital-Based Retrospective Observational Study	Anesthesia: Essays and Researches	16	3	296-300	RW
Lapadula 2020	Remdesivir Use in Patients Requiring Mechanical Ventilation due to COVID-19	Open Forum Infectious Diseases	7	11	-	RW
Leding 2023	Treatment effect modifiers in hospitalised patients with COVID-19 receiving remdesivir and dexamethasone	Infectious Diseases	55	5	351-360	RW
Leegwater 2023	Rapid Response to Remdesivir in Hospitalised COVID-19 Patients: A Propensity Score Weighted Multicentre Cohort Study	Infectious Diseases and Therapy	12	-	2471-2484	RW

Author, year	Title	Journal	Volume	Issue	Page	Study type
Lim 2021	Lower Risk of ICU Admission with Remdesivir in Patients Hospitalized with COVID-19 Pneumonia	Open Forum Infectious Diseases	8	Suppl 1	S364	RW
Lim 2022	Remdesivir in the Treatment of COVID-19: A Propensity Score-Matched Analysis from a Public Hospital in New York City Assessing Renal and Hepatic Safety	Journal of Clinical Medicine	11	11	3132	RW
Lucijanic 2022	Real-life experience with remdesivir for treatment of hospitalized coronavirus disease 2019 patients: matched case-control study from a large tertiary hospital registry	Croatian Medical Journal	63	6	536-543	RW
Mahajan 2021	Clinical outcomes of using remdesivir in patients with moderate to severe COVID-19: A prospective randomised study	Indian Journal of Anaesthesia	65	SUPPL 1	S41–S46	RCT
Malik 2022	The utility of remdesivir in SARS-CoV-2: A single tertiary care center experience from a developing country	Exploratory Research in Clinical and Social Pharmacy	5	-	100107	RW
Marocco 2023	Use of Remdesivir in Patients with SARS-CoV-2 Pneumonia in a Real-Life Setting during the Second and Third COVID-19 Epidemic Waves	Viruses	15	4	947	RW
Marx 2022	Clinical outcomes of hospitalized COVID-19 patients treated with remdesivir: a retrospective analysis of a large tertiary care center in Germany	Infection.	-	-	-	RW
Metchurtchlishvili 2023	Effect of remdesivir on mortality and the need for mechanical ventilation among hospitalized patients with COVID-19: real-world data from a resource-limited country	International Journal of Infectious Diseases	129	-	63–69	RW
Mohanty 2021	Success rate of remdesivir, convalescent plasma, and tocilizumab in moderate to severe Covid-19 pneumonia: our experience in a tertiary care center	Journal of Family Medicine and Primary Care	10	NR	4236-4241	RW
Monardo 2022	Real-life use of high-dose Anakinra in Covid-19 patients treated with remdesivir	CROI 2022	-	-	-	RW
Mozaffari 2021a	Remdesivir Treatment in Patients Hospitalized with COVID-19: a Comparative Analysis of In-Hospital All-Cause Mortality	Open forum infectious diseases	8	Suppl 1	S27-S28	RW

Author, year	Title	Journal	Volume	Issue	Page	Study type
Mozaffari 2021b	Early treatment with remdesivir and in-hospital mortality among hospitalised COVID-19 patients in the real-world setting	ECCMID 2021	-	-	-	RW
Mozaffari 2022	Remdesivir Treatment in Hospitalized Patients With Coronavirus Disease 2019 (COVID-19): A Comparative Analysis of In-hospital All-cause Mortality in a Large Multicenter Observational Cohort	Clinical Infectious Diseases	75	1	e450-e458	RW
Mozaffari 2023a	Remdesivir Is Associated With Reduced Mortality in COVID-19 Patients Requiring Supplemental Oxygen Including Invasive Mechanical Ventilation Across SARS-CoV-2 Variants	Open Forum Infectious Diseases	10	10	-	RW
Mozaffari 2023b	Remdesivir is associated with reduced readmission after COVID-19 hospitalization	CROI 2023	-	-	-	RW
Nevalainen 2022	Effect of Remdesivir on Recovery, Quality of Life, and Long-COVID Symptoms One Year after Hospitalization for COVID-19 Infection: A Randomized Controlled SOLIDARITY Finland Trial	Open Forum Infectious Diseases	9	SUPPL 2	S480	RCT
Ngo 2022	A Retrospective Study of Dexamethasone, Remdesivir, and Baricitinib in Severe COVID-19	Canadian Journal of Infectious Diseases and Medical Microbiology	2022	-	9209618	RW
Panda 2022	Effect of Remdesivir Administration on Occurrence of Major Adverse Cardiac Events in Critically Ill COVID-19 Pneumonia: A Retrospective Observational Study	Indian Journal of Critical Care Medicine	26	9	993–999	RW
Pantazopoulos 2022	Remdesivir-induced Bradycardia is not Associated with Worse Outcome in Patients with COVID-19: A Retrospective Analysis	American Journal of Cardiovascular Drugs	22	6	705–710	RW
Paranjape 2021	Early Use of Remdesivir in Patients Hospitalized with COVID-19 Improves Clinical Outcomes: A Retrospective Observational Study	Infectious Diseases in Clinical Practice	29	5	e282–e286	RW
Petrakis 2022	Greek Remdesivir Cohort (GREC) Study: Effectiveness of Antiviral Drug Remdesivir in Hospitalized Patients with COVID-19 Pneumonia	Microorganisms	10	10	1949	RW

Author, year	Title	Journal	Volume	Issue	Page	Study type
Pham 2023	Clinical use of remdesivir in COVID-19 treatment: A retrospective cohort study	BMJ Open	13	6	e070489	RW
Pivato 2022	Real-Life Use of Remdesivir in Hospitalised Covid-19 Patients with Severe Pneumonia: An Observational Study from an Italian University Hospital	European Journal of Hospital Pharmacy	29	Suppl 1	A176	RW
Platzer 2023	The Effect of Early Remdesivir Administration in COVID-19 Disease Progression	IDWeek 2023	-	-	-	RW
Poliseno 2021	Efficacy and safety of remdesivir over two waves of the sars-cov-2 pandemic	Antibiotics	10	12	-	RW
Polizzotto 2022	Hyperimmune immunoglobulin for hospitalised patients with COVID-19 (ITAC): a double-blind, placebo-controlled, phase 3, randomised trial	Lancet	399	10324	530-540	RCT
Rai 2022	Effectiveness of remdesivir on hospital stay duration and mortality in patients with COVID-19 infection: A single-center retrospective observational study	Lung India	39	1	39	RW
Rawat 2022	RWD31 Comparison of Remdesivir As Monotherapy or in Combination with Steroids in Treating COVID-19 Patients	Value in Health	25	7 Supplement	S581-S582	RW
Razzaq 2022	Comparison of Clinical Improvement with VS without Remdesivir Treatment in Hospitalized Patients with COVID-19	Medical Forum Monthly	33	12	14–18	RW
Read 2023	Decision Making and the Questions Raised When Real World Evidence From a Federated Data Network Conflicts With RCT Data: A Use Case on COVID-19 Treatments	ISPOR Europe 2023 2023	-	-	-	RW
Rivera 2023	Clinical outcomes of COVID-19 treated with remdesivir across the continuum of care	Influenza & Other Respiratory Viruses	17	5	e13136	RW
Rosas 2021	Tocilizumab and remdesivir in hospitalized patients with severe COVID-19 pneumonia: a randomized clinical trial	Intensive care medicine	47	11	1258–1270	RCT
Russo 2021	Efficacy of remdesivir-containing therapy in hospitalized COVID-19 patients: a prospective clinical experience	Journal of Clinical Medicine	10	17	3784	RW

Author, year	Title	Journal	Volume	Issue	Page	Study type
Russo 2022	Mortality in SARS-CoV-2 Hospitalized Patients Treated with Remdesivir: A Nationwide, Registry-Based Study in Italy RW	Viruses	14	6	-	RW
Said 2023	Remdesivir Use in Hospitalized Covid-19 Patients	Critical Care Medicine	51	Suppl 1	208	RW
Sattoju 2023	Dexamethasone, dexamethasone + remdesivir in treating moderate to severe COVID-19: retrospective observational cohort study	Journal of Infection in Developing Countries	17	7	953-960	RW
Sellers 2023	Patients with moderate to severe COVID-19 outcomes on remdesivir according to baseline 4C mortality score	Pulmonary Pharmacology & Therapeutics	78	-	102188	RW
Senthiappan 2023	Clinical Outcomes of Remdesivir in Moderate and Severe Cases of COVID-19: A Retrospective Cohort Study	Journal of Clinical and Diagnostic Research	17	1	OC36-OC38	RW
So 2021	Use of Baricitinib in Combination With Remdesivir and Steroid in COVID-19 Treatment: A Multicenter Retrospective Study	Cureus	13	12	e20620	RW
Spagnuolo 2022	Impact of Remdesivir on SARS-CoV-2 Clearance in a Real-Life Setting: A Matched-Cohort Study	Drug Design, Development and Therapy	16	-	3645-3654	RW
Spinner 2020	Effect Of Remdesivir Vs Standard Care On Clinical Status At 11 Days In Patients With Moderate Covid-19: A Randomized Clinical Trial.$	JAMA - Journal of the American Medical Association	324	11	1048-1057	RCT
Temesgen 2021	Lenzilumab Efficacy and Safety in Newly Hospitalized COVID-19 Subjects: Results from the LIVE-AIR Phase 3 Randomized Double-Blind Placebo-Controlled Trial	The Lancet	10	3	237–246	RCT
Ughi 2023	Effectiveness and Safety of Remdesivir in Treating Hospitalised Patients with COVID-19: A Propensity Score Analysis of Real-Life Data from a Monocentric Observational Study in Times of Health Emergency	Clinical Drug Investigation	43	10	763-771	RW
Umeh 2023	Bradycardia and Outcomes in COVID-19 Patients on Remdesivir: A Multicenter Retrospective Study	Cardiology Research	14	3	192–200	RW

Author, year	Title	Journal	Volume	Issue	Page	Study type
Viray 2023	Clinical Effectiveness and Adverse Events of Adjunctive Remdesivir Administration Vs Standard of Care in Adults with Severe and Critical Covid-19	International Journal of Infectious Diseases	130	Suppl 2	S143	RW
Wang 2020	Remdesivir in adults with severe COVID-19: a randomised, double-blind, placebo-controlled, multicentre trial	The Lancet	395	10236	1694	RCT
WHO Solidarity 2021	Repurposed Antiviral Drugs for COVID-19 – Interim WHO Solidarity Trial Results	New England Journal of Medicine	384	-	497–511	RCT
Williams 2023	Real World Evaluation of Clinical Outcomes for Short-Duration Remdesivir Therapy in Hospitalized Patients with Mild-Moderate COVID-19	IDWeek 2023	-	-	-	RW
Wolfe 2022	Baricitinib versus dexamethasone for adults hospitalized with COVID-19 (ACTT-4): a randomized, double-blind, double placebo-controlled trial	The Lancet Respiratory Medicine	10	9	888–899	RCT
Wong 2022a	Optimal Timing of Remdesivir Initiation in Hospitalized Patients with Coronavirus Disease 2019 (COVID-19) Administered with Dexamethasone	Clinical Infectious Diseases	75	1	E499-E508	RW
Wong 2022b	Clinical improvement, outcomes, antiviral activity, and costs associated with early treatment with remdesivir for patients with Covid-19	Clinical Infectious Diseases	74	8	1450-1458	RW
Wu 2022	Acute Kidney Injury Associated With Remdesivir: A Comprehensive Pharmacovigilance Analysis of COVID-19 Reports in FAERS	Frontiers in Pharmacology	13	-	692828	RW

Supplemental Figure S1. SLR process

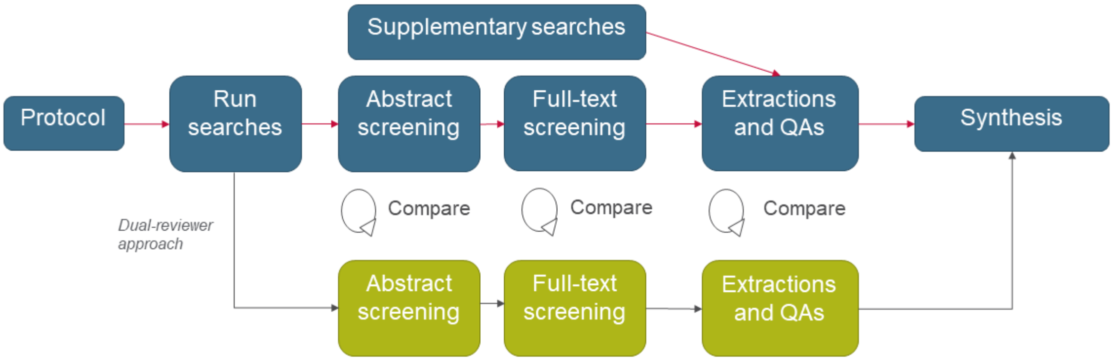

Abbreviations: QA, quality assessment; RCT, randomized controlled trial; SLR, systematic literature review.

Supplemental Figure S2. Study regions

A. **RW studies**

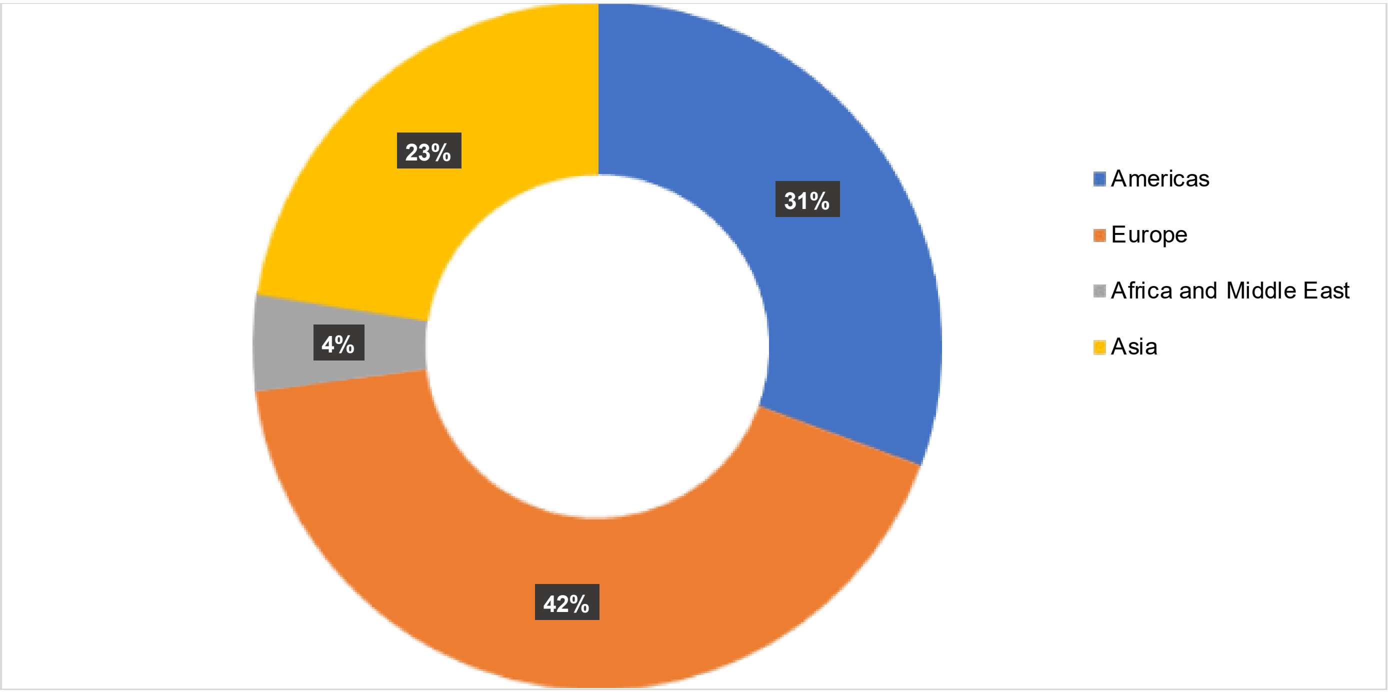

B. **RCTs**

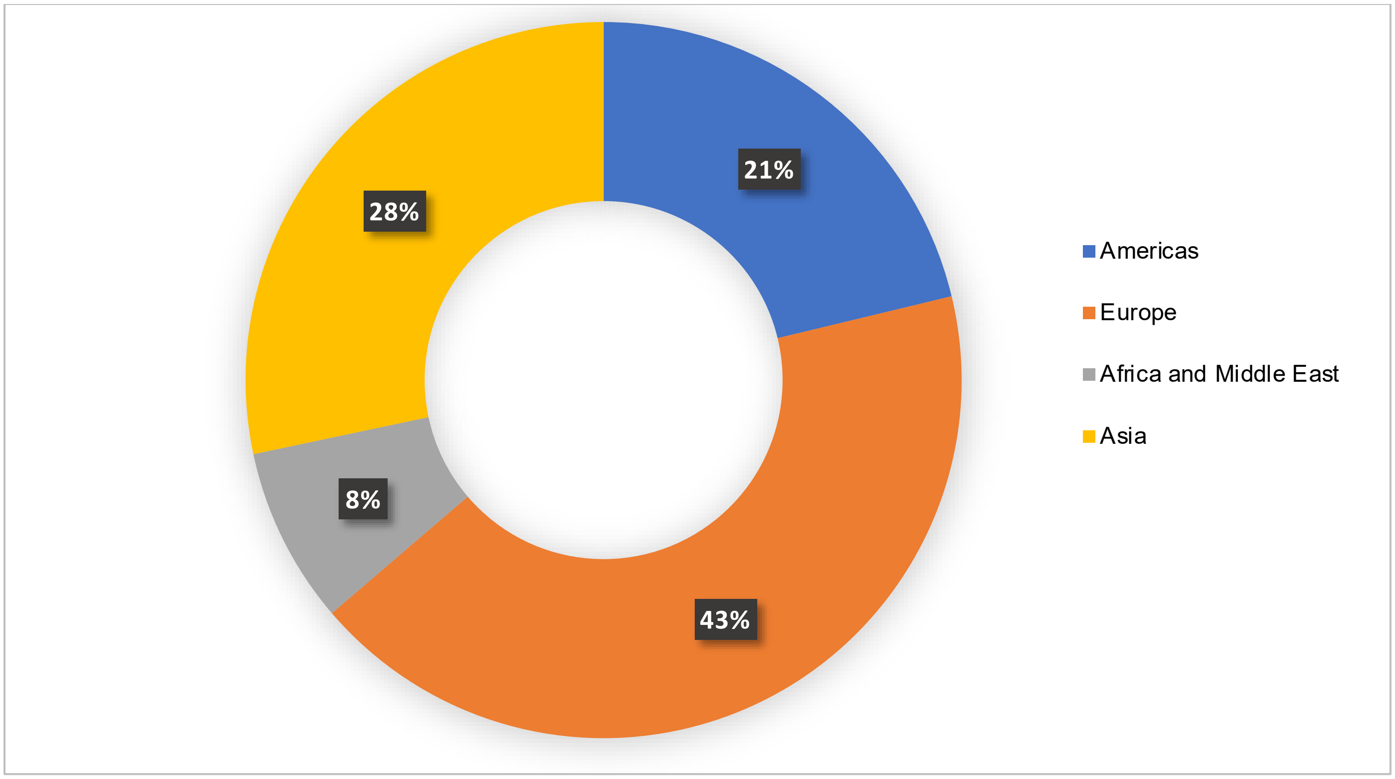

(A) Geography of participated in observational studies countries; (B) Geography of participated in RCTs countries.

<u>Supplemental Figure S3.</u> Forest plot for time to clinical improvement in hospitalized COVID-19 patients, overall population

Study	Design	N	%Not requiring oxygen	Treatment duration (days)	Time to clinical improvement	MD (95% CI)
ACTT-1, Beigel 2020	RCT	1062	13%	10		-4.00 (-4.15 to -3.85)
DisCoVeRy, Ader 2021*	RCT	832		5-10		0.00 (-2.23 to 2.18)
Wang 2020*	RCT	236	2%	10		-2.00 (-6.27 to 2.19)
**Sub-summary (RCT) (tau=0, p<0.001)**						**-2.98 (-3.01 to -1.09)**
Wong 2022a	Retrospective database study	1544	73.8%	5-10		-1.00 (-2.30 to 0.32)
Garibaldi 2022*	Retrospective study	36656	14.6%	5		-2.00 (-2.27 to -1.73)
Viray 2023	Retrospective study	318				-5.00 (-7.02 to -3.04)
**Sub-summary (RWS) (tau=0.171, p=0.002)**						**-2.20 (-3.43 to -0.67)**
**Total summary (tau=0.158, p=0.001)**						**-1.84 (-2.81 to -0.69)**

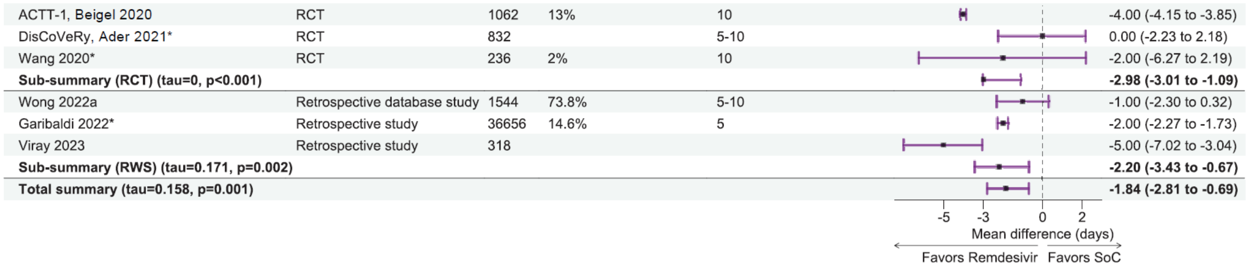

* Corticosteroids were used in both remdesivir and no remdesivir groups.
MD, mean difference; RCT, randomized controlled trial; RWS, real world study; SoC, standard of care.
Note: The MDs reported in the plot resulted from the meta-analysis based on the number of events reported in the original studies.

Supplemental Figure S4. Forest plot for risk of rehospitalization in hospitalized COVID-19 patients, overall population

Study	Design	N	%Not requiring oxygen	Treatment duration (days)	Rehospitalization	OR (95% CI)
ACTT-1, Beigel 2020	RCT	1062	13%	10		1.66 (0.88 to 3.11)
Finn 2022	Prospective cohort study	2111				0.68 (0.50 to 0.92)
Caffrey 2023*	Retrospective study	16416	16.8%			0.66 (0.59 to 0.73)
Mozaffari 2023 Delta period (May21-Nov21)	Retrospective study	127393	41%			0.63 (0.61 to 0.66)
Mozaffari 2023 Omicron period (Dec21-Apr22)	Retrospective study	81300	41%			0.87 (0.83 to 0.91)
Mozaffari 2023 Pre-delta period (Dec20-Apr21)	Retrospective study	231908	41%			0.66 (0.64 to 0.68)
**Sub-summary (RWS) (tau=0.11, p<0.001)**						**0.70 (0.63 to 0.78)**
**Total summary (tau=0.11, p<0.001)**						**0.72 (0.64 to 0.81)**

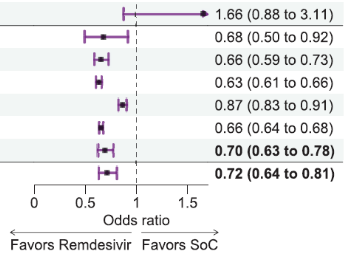

* Corticosteroids were used in both remdesivir and no remdesivir groups.
OR, odds ratio; RCT, randomized controlled trial; RWS, real world study; SoC, standard of care.
Note: The ORs reported in the plot resulted from the meta-analysis based on the number of events reported in the original studies.

References

1. National Institutes of Health. Therapeutic Management of Hospitalized Adults With COVID-19. 2024. Accessed 03/01/24. https://www.covid19treatmentguidelines.nih.gov/management/clinical-management-of-adults/hospitalized-adults--therapeutic-management/
2. Bartoletti M, Azap O, Barac A, et al. European society of clinical microbiology and infectious diseases guidelines for coronavirus disease 2019: an update on treatment of patients with mild/moderate disease. *Clin Microbiol Infect*. Dec 2022;28(12):1578-1590. doi:10.1016/j.cmi.2022.08.013
3. Bartoletti M, Azap O, Barac A, et al. ESCMID COVID-19 living guidelines: drug treatment and clinical management. *Clin Microbiol Infect*. Feb 2022;28(2):222-238. doi:10.1016/j.cmi.2021.11.007
4. Infectious Diseases Society of America. IDSA Guidelines on the Treatment and Management of Patients with COVID-19. 2023. Accessed 01/12/24. https://www.idsociety.org/practice-guideline/covid-19-guideline-treatment-and-management/
5. World Health Organization. Therapeutics and COVID-19: living guideline. 2023. Accessed 01/12/24. https://iris.who.int/bitstream/handle/10665/365584/WHO-2019-nCoV-therapeutics-2023.1-eng.pdf?sequence=1
6. Chalmers JD, Crichton ML, Goeminne PC, et al. Management of hospitalised adults with coronavirus disease 2019 (COVID-19): a European Respiratory Society living guideline. *Eur Respir J*. Apr 2021;57(4)doi:10.1183/13993003.00048-2021
7. Crichton ML, Goeminne PC, Tuand K, et al. The impact of therapeutics on mortality in hospitalised patients with COVID-19: systematic review and meta-analyses informing the European Respiratory Society living guideline. *Eur Respir Rev*. Dec 31 2021;30(162)doi:10.1183/16000617.0171-2021
8. Roche N, Crichton ML, Goeminne PC, et al. Update June 2022: management of hospitalised adults with coronavirus disease 2019 (COVID-19): a European Respiratory Society living guideline. *Eur Respir J*. Aug 2022;60(2)doi:10.1183/13993003.00803-2022
9. Beigel JH, Tomashek KM, Dodd LE, et al. Remdesivir for the Treatment of Covid-19 - Final Report. *N Engl J Med*. Nov 5 2020;383(19):1813-1826. doi:10.1056/NEJMoa2007764
10. Kalil AC, Patterson TF, Mehta AK, et al. Baricitinib plus Remdesivir for Hospitalized Adults with Covid-19. Journal article. *New England journal of medicine*. 2021;384(9):795 - 807. doi:10.1056/NEJMoa2031994

11. Kalil AC, Mehta AK, Patterson TF, et al. Efficacy of interferon beta-1a plus remdesivir compared with remdesivir alone in hospitalised adults with COVID-19: a double-bind, randomised, placebo-controlled, phase 3 trial. *The Lancet Respiratory Medicine*. 12 2021;9(12):1365-1376. doi:https://dx.doi.org/10.1016/S2213-2600(21)00384-2
12. Wolfe CR, Tomashek KM, Patterson TF, et al. Baricitinib versus dexamethasone for adults hospitalised with COVID-19 (ACTT-4): a randomised, double-blind, double placebo-controlled trial. Journal article. *Lancet respiratory medicine*. 2022;10(9):888 - 899. doi:10.1016/S2213-2600(22)00088-1
13. Ali K, Azher T, Baqi M, et al. Remdesivir for the treatment of patients in hospital with COVID-19 in Canada: a randomized controlled trial. *CMAJ Canadian Medical Association Journal*. 02 22 2022;194(7):E242-E251. doi:https://dx.doi.org/10.1503/cmaj.211698
14. Ader F, Bouscambert-Duchamp M, Hites M, et al. Remdesivir plus standard of care versus standard of care alone for the treatment of patients admitted to hospital with COVID-19 (DisCoVeRy): a phase 3, randomised, controlled, open-label trial. *Lancet Infect Dis*. Feb 2022;22(2):209-221. doi:10.1016/s1473-3099(21)00485-0
15. Hormati A, Ahmadpour S, Ghadir MR, et al. Comparison of Efficacy of Remdesivir with Supportive Care Alone in the Treatment of Critically Sick Adult and Child COVID-19 Patients: A Randomized Clinical Trial. *Anti-Infective Agents*. February 2023;21(1) (no pagination)e170522204883. doi:https://dx.doi.org/10.2174/2211352520666220517092803
16. Polizzotto MN, Nordwall J, Babiker AG, et al. Hyperimmune immunoglobulin for hospitalised patients with COVID-19 (ITAC): a double-blind, placebo-controlled, phase 3, randomised trial. *The Lancet*. 05 Feb 2022;399(10324):530-540. doi:https://dx.doi.org/10.1016/S0140-6736%2822%2900101-5
17. Temesgen Z, Burger CD, Baker J, et al. Lenzilumab Efficacy and Safety in Newly Hospitalized Covid-19 Subjects: Results from the Live-Air Phase 3 Randomized Double-Blind Placebo-Controlled Trial. Preprint. *MedRxiv : the Preprint Server for Health Sciences*. May 05 2021;05:05. doi:https://dx.doi.org/10.1101/2021.05.01.21256470
18. Mahajan L, Singh AP, Gifty. Clinical outcomes of using remdesivir in patients with moderate to severe COVID-19: A prospective randomised study. *Indian Journal of Anaesthesia*. March 2021;65(13 Supplement 1):S41-S46. doi:https://dx.doi.org/10.4103/ija.IJA_149_21
19. Barratt-Due A, Olsen IC, Nezvalova-Henriksen K, et al. Evaluation of the Effects of Remdesivir and Hydroxychloroquine on Viral Clearance in COVID-19 : A Randomized Trial. *Annals of Internal Medicine*. 09 2021;174(9):1261-1269. doi:https://dx.doi.org/10.7326/M21-0653
20. Jittamala P, Schilling WHK, Watson JA, et al. Clinical Antiviral Efficacy of Remdesivir in Coronavirus Disease 2019: An Open-Label, Randomized Controlled Adaptive Platform Trial (PLATCOV). *Journal of Infectious Diseases*. 11 11 2023;228(10):1318-1325. doi:https://dx.doi.org/10.1093/infdis/jiad275
21. Rosas IO, Diaz G, Gottlieb RL, et al. Tocilizumab and remdesivir in hospitalized patients with severe COVID-19 pneumonia: a randomized clinical trial. *Intensive Care Medicine*. 11 2021;47(11):1258-1270. doi:https://dx.doi.org/10.1007/s00134-021-06507-x
22. Spinner CD, Gottlieb RL, Criner GJ, et al. Effect of Remdesivir vs Standard Care on Clinical Status at 11 Days in Patients With Moderate COVID-19: A Randomized Clinical Trial. *JAMA*. 09 15 2020;324(11):1048-1057. doi:https://dx.doi.org/10.1001/jama.2020.16349
23. Goldman JD, Lye DCB, Hui DS, et al. Remdesivir for 5 or 10 Days in Patients with Severe Covid-19. Clinical Trial, Phase III Comparative Study Randomized Controlled Trial Research Support, Non-U.S. Gov't. *New England Journal of Medicine*. 11 05 2020;383(19):1827-1837. doi:https://dx.doi.org/10.1056/NEJMoa2015301

24. Wang Y, Zhang D, Du G, et al. Remdesivir in adults with severe COVID-19: a randomised, double-blind, placebo-controlled, multicentre trial. Journal article. *Lancet (london, england)*. 2020;395(10236):1569 - 1578. doi:10.1016/S0140-6736(20)31022-9
25. Acharya TA, Joshi KJ, Patel DD, Shah SN, Mehta DS. Study of Clinical Outcome and Healthcare Modalities of COVID-19 Patients Treated With Remdesivir at a Tertiary Care Teaching Hospital. *Cureus*. Jan 2022;14(1):e21535. doi:https://dx.doi.org/10.7759/cureus.21535
26. Aiello TF, Puerta-Alcalde P, Chumbita M, et al. Current outcomes of SARS-CoV-2 Omicron variant infection in high-risk haematological patients treated early with antivirals. *Journal of Antimicrobial Chemotherapy*. 06 01 2023;78(6):1454-1459. doi:https://dx.doi.org/10.1093/jac/dkad105
27. Aksak-Was BJ, Chober D, Serwin K, et al. Remdesivir Reduces Mortality in Hemato-Oncology Patients with COVID-19. *Journal of Inflammation Research*. 2022;15:4907-4920. doi:https://dx.doi.org/10.2147/JIR.S378347
28. Alexander H, Gunasekaran K, John JS, et al. Evaluation of Remdesivir to the outcomes of hospitalized patients with COVID-19 infection in a tertiary-care hospital in southern India. *Journal of Infection in Developing Countries*. March 2023;17(3):304-310. doi:https://dx.doi.org/10.3855/JIDC.16642
29. Alshamrani AA, Assiri AM, Almohammed OA. Comprehensive evaluation of six interventions for hospitalized patients with COVID-19: A propensity score matching study. *Saudi Pharmaceutical Journal*. Apr 2023;31(4):517-525. doi:https://dx.doi.org/10.1016/j.jsps.2023.02.004
30. Arribas Lopez JR, Ruiz Seco MP, Losa FF, et al. Remdesivir associated with decreased mortality in hospitalised COVID-19 patients: a real-world evidence study using natural language processing. Conference Abstract. 2023;
31. Attena E, Caturano A, Annunziata A, et al. Remdesivir treatment and clinical outcome in non-severe hospitalized COVID-19 patients: a propensity score matching multicenter Italian hospital experience. Multicenter Study. *European Journal of Clinical Pharmacology*. Jul 2023;79(7):967-974. doi:https://dx.doi.org/10.1007/s00228-023-03499-z
32. Ayodele O, Ren K, Zhao J, et al. Real-world treatment patterns and clinical outcomes for inpatients with COVID-19 in the US from September 2020 to February 2021. Research Support, Non-U.S. Gov't. *PLoS ONE [Electronic Resource]*. 2021;16(12):e0261707. doi:https://dx.doi.org/10.1371/journal.pone.0261707
33. Bansal S, Kalpakam H, Bysani S, Varsha A, Mehta RM. A shorter symptom onset to remdesivir treatment (SORT) interval is associated with a lower mortality in moderate to severe COVID-19: A real-world analysis. Conference Abstract. *American Journal of Respiratory and Critical Care Medicine Conference: American Thoracic Society International Conference, ATS*. 2021;203(9)doi:https://dx.doi.org/10.1164/ajrccm-conference.2021.203.1_MeetingAbstracts.A3811
34. Bárczi E, Polivka L, Nagy A, Horváth G, Müller V. Formerly hospitalized patients treated with remdesivir presented less respiratory post-COVID symptoms. *European Respiratory Journal*. 2023;62(suppl 67):PA296. doi:10.1183/13993003.congress-2023.PA296
35. Basoulis D, Voutsinas PM, Samara S, et al. Safety and effectiveness of three-day remdesivir regimen to prevent progression to severe COVID-19: a single-centre, real-world study. presented at: ECCMID 2023;
36. Bavaro DF, Diella L, Belati A, et al. Efficacy of Remdesivir and Neutralizing Monoclonal Antibodies in Monotherapy or Combination Therapy in Reducing the Risk of Disease Progression in Elderly or Immunocompromised Hosts Hospitalized for COVID-19: A Single Center Retrospective Study. Research Support, Non-U.S. Gov't. *Viruses*. 05 19 2023;15(5):19. doi:https://dx.doi.org/10.3390/v15051199
37. Bechman K, Yates M, Mann K, et al. Inpatient COVID-19 mortality has reduced over time: Results from an observational cohort. *PLoS ONE [Electronic Resource]*. 2022;17(1):e0261142. doi:https://dx.doi.org/10.1371/journal.pone.0261142

38. Behboodikhah H, Shorafa E, Karimzadeh I, et al. Evaluation of the Costs and Outcomes of COVID-19 Therapeutic Regimens in Hospitalized Patients in Shiraz. *Iranian Journal of Science & Technology Transaction a Science*. 2022;46(5):1339-1347. doi:https://dx.doi.org/10.1007/s40995-022-01351-0
39. Benfield T, Bodilsen J, Brieghel C, et al. Improved Survival Among Hospitalized Patients With Coronavirus Disease 2019 (COVID-19) Treated With Remdesivir and Dexamethasone. A Nationwide Population-Based Cohort Study. *Clinical Infectious Diseases*. 12 06 2021;73(11):2031-2036. doi:https://dx.doi.org/10.1093/cid/ciab536
40. Bernal E, Garcia-Villalba E, Pons E, et al. Remdesivir plus dexamethasone is associated to improvement in the clinical outcome of COVID-19 hospitalized patients regardless of their vaccination status. Observational Study. *Medicina Clinica*. 08 25 2023;161(4):139-146. doi:https://dx.doi.org/10.1016/j.medcli.2023.03.025
41. Bistrovic P, Manola S, Lucijanic M. Bradycardia during remdesivir treatment might be associated with improved survival in patients with COVID-19: a retrospective cohort study on 473 patients from a tertiary centre. *Postgraduate Medical Journal*. 07 2022;98(1161):501-502. doi:https://dx.doi.org/10.1136/postgradmedj-2021-141079
42. Boglione L, Dodaro V, Meli G, et al. Remdesivir treatment in hospitalized patients affected by COVID-19 pneumonia: A case-control study. Observational Study. *Journal of Medical Virology*. 08 2022;94(8):3653-3660. doi:https://dx.doi.org/10.1002/jmv.27768
43. Breskin A, Wiener C, Adimora AA, et al. Effectiveness of Remdesivir Treatment Protocols Among Patients Hospitalized with COVID-19: A Target Trial Emulation. Research Support, Non-U.S. Gov't. *Epidemiology*. 05 01 2023;34(3):365-375. doi:https://dx.doi.org/10.1097/EDE.0000000000001598
44. Burhan E, Syahruddin E, Isbaniah F, et al. Evaluation of safety and effectiveness of remdesivir in treating COVID-19 patients after emergency use authorization study. *Frontiers in Pharmacology*. 2023;14:1205238. doi:https://dx.doi.org/10.3389/fphar.2023.1205238
45. Butt A, Talisa V, Yan P, Shaikh O, Mayr FG. Risk of Death in Remdesivir Treated and Untreated Patients with Covid-19 Infection. Conference Abstract. *Topics in Antiviral Medicine*. April 2023;31(2):220.
46. Caffrey A, Liao JX, Lopes V. Utilization of Remdesivir for COVID-19 in the National Veterans Affairs Healthcare System. Conference Abstract. *Open Forum Infectious Diseases*. November 2021;8(SUPPL 1):S380. doi:https://dx.doi.org/10.1093/ofid/ofab466.753
47. Caffrey AR, Liao JX, Lopes VV, LaPlante KL, Appaneal HJ. Real-world safety and effectiveness of remdesivir and corticosteroids in hospitalized patients with COVID-19. *Covid*. 2023;3(2):198-217.
48. Ahmed A, Rojo P, Agwu A, et al. REMDESIVIR TREATMENT for COVID-19 in HOSPITALIZED CHILDREN: CARAVAN INTERIM RESULTS. Conference Abstract. *Topics in Antiviral Medicine*. March 2022;30(1 SUPPL):296-297.
49. Chaudhary BR, Dudhrejia PJ, Gambhir RM, Rathod MM. Study of Efficacy of Injection Remdesivir in Patients of COVID-19. *Journal of the Association of Physicians of India*. Apr 2023;71(4):11-12. doi:https://dx.doi.org/10.5005/japi-11001-0217
50. Chavalertsakul K, Petnak T, Boonsarngsuk V, et al. Remdesivir versus favipiravir in hospitalized patients with moderate COVID-19 pneumonia: A propensity score-matched retrospective cohort study. *European Respiratory Journal*. 2023;62(suppl 67):PA1686. doi:10.1183/13993003.congress-2023.PA1686
51. Chokkalingam AP, Hayden J, Goldman JD, et al. Association of Remdesivir Treatment With Mortality Among Hospitalized Adults With COVID-19 in the United States. *JAMA Network Open*. 12 01 2022;5(12):e2244505. doi:https://dx.doi.org/10.1001/jamanetworkopen.2022.44505
52. Cilloniz C, Motos A, Castaneda T, Gabarrus A, Barbe F, Torres A. Remdesivir and survival outcomes in critically ill patients with COVID-19: A multicentre observational cohort study. *Journal of Infection*. 03 2023;86(3):256-308. doi:https://dx.doi.org/10.1016/j.jinf.2022.12.027
53. Das D, Mukhopadhyay P, Banerjee D. Mortality comparision in patients receiving either Remdesivir or Remdesivir plus Baricitinib combination in case of moderate to severe COVID-19

Pneumonia: A retrospective study. *Journal of the Association of Physicians of India*. Apr 2022;70(4):11-12.
54. De Vito A, Poliseno M, Zauli B, et al. Efficacy of remdesivir in reducing mortality in people hospitalised for SARS-CoV-2 infection: a real-life experience. presented at: ECCMID 2022;
55. De Vito A, Bitti A, Colpani A, et al. Safety and efficacy of remdesivir three-day course in SARS-CoV-2-infected patients: a real-life experience. presented at: ECCMID 2023;
56. Delgado AC, Cornett B, Choi YJ, et al. Investigational medications in 9,638 hospitalized patients with severe COVID-19: lessons from the "fail-and-learn" strategy during the first two waves of the pandemic in 2020. *Patient Safety in Surgery [Electronic Resource]*. Apr 11 2023;17(1):7. doi:https://dx.doi.org/10.1186/s13037-023-00358-9
57. Devgun JM, Zhang R, Brent J, et al. Identification of Bradycardia Following Remdesivir Administration Through the US Food and Drug Administration American College of Medical Toxicology COVID-19 Toxic Pharmacovigilance Project. Multicenter Study

Research Support, U.S. Gov't, P.H.S. *JAMA Network Open*. 02 01 2023;6(2):e2255815. doi:https://dx.doi.org/10.1001/jamanetworkopen.2022.55815
58. Diaz GA, Christensen AB, Pusch T, et al. Remdesivir and Mortality in Patients With Coronavirus Disease 2019. Research Support, Non-U.S. Gov't. *Clinical Infectious Diseases*. 05 30 2022;74(10):1812-1820. doi:https://dx.doi.org/10.1093/cid/ciab698
59. Dobrowolska K, Zarebska-Michaluk D, Brzdek M, et al. Retrospective Analysis of the Effectiveness of Remdesivir in COVID-19 Treatment during Periods Dominated by Delta and Omicron SARS-CoV-2 Variants in Clinical Settings. *Journal of Clinical Medicine*. Mar 19 2023;12(6):19. doi:https://dx.doi.org/10.3390/jcm12062371
60. Elshaboury RH, Adamsick ML, Huang J, et al. Short vs. Long Symptom Duration Prior to Remdesivir for Hospitalized Patients with COVID-19. Conference Abstract. *Open Forum Infectious Diseases*. December 2022;9(Supplement 2):S495-S496. doi:https://dx.doi.org/10.1093/ofid/ofac492.993
61. Estill J, Venkova-Marchevska P, Gunthard HF, et al. Treatment effect of remdesivir on the mortality of hospitalised COVID-19 patients in Switzerland across different patient groups: a tree-based model analysis. *Swiss Medical Weekly*. August 2023;153(8) (no pagination)40095. doi:https://dx.doi.org/10.57187/smw.2023.40095
62. Falcone M, Suardi LR, Tiseo G, et al. Early Use of Remdesivir and Risk of Disease Progression in Hospitalized Patients With Mild to Moderate COVID-19. Observational Study

Research Support, Non-U.S. Gov't. *Clinical Therapeutics*. 03 2022;44(3):364-373. doi:https://dx.doi.org/10.1016/j.clinthera.2022.01.007
63. Finn A, Jindal A, Andrea SB, Selvaraj V, Dapaah-Afriyie K. Association of Treatment with Remdesivir and 30-day Hospital Readmissions in Patients Hospitalized with COVID-19. Multicenter Study. *American Journal of the Medical Sciences*. 05 2022;363(5):403-410. doi:https://dx.doi.org/10.1016/j.amjms.2022.01.021
64. Garibaldi BT, Wang K, Robinson ML, et al. Real-World Effectiveness of Remdesivir in Adults Hospitalized With Coronavirus Disease 2019 (COVID-19): A Retrospective, Multicenter Comparative Effectiveness Study. *Clinical Infectious Diseases*. 08 24 2022;75(1):e516-e524. doi:https://dx.doi.org/10.1093/cid/ciab1035
65. Gragera Gomez M, Redondo Galan C, Rivas Rodriguez MD, Rojas Albarran A, Gil Garcia A, Rangel Mayoral JF. Experience of Baricitinib-Remdesivir Use in Patients with Sars-Cov-2 Infection. Conference Abstract. *European Journal of Hospital Pharmacy*. March 2022;29(SUPPL 1):A157. doi:https://dx.doi.org/10.1136/ejhpharm-2022-eahp.330

66. Petrakis V, Rapti V, Akinosoglou K, et al. Greek Remdesivir Cohort (GREC) Study: Effectiveness of Antiviral Drug Remdesivir in Hospitalized Patients with COVID-19 Pneumonia. *Microorganisms*. Sep 30 2022;10(10):30. doi:https://dx.doi.org/10.3390/microorganisms10101949
67. Grundmann A, Wu CH, Hardwick M, et al. Fewer COVID-19 Neurological Complications with Dexamethasone and Remdesivir. *Annals of Neurology*. January 2023;93(1):88-102. doi:https://dx.doi.org/10.1002/ana.26536
68. Gunasekaran K, John JS, Alexander H, Gracelin N, Samuel P, Rupali P. Does Remdesivir Impact the Clinical Outcome of Patients with COVID 19 Infection? Conference Abstract. *Open Forum Infectious Diseases*. November 2021;8(SUPPL 1):S371-S372. doi:https://dx.doi.org/10.1093/ofid/ofab466.739
69. Jeyapalina S, Lundquist M, Wei G, Stoddard G, Agarwal J. Effectiveness of Remdesivir as Treatment for COVID-19 Positive US Veterans. Conference Abstract. *Open Forum Infectious Diseases*. December 2022;9(Supplement 2):S480-S481. doi:https://dx.doi.org/10.1093/ofid/ofac492.966
70. Karolyi M, Kaltenegger L, Pawelka E, et al. Early administration of remdesivir may reduce mortality in hospitalized COVID-19 patients : A propensity score matched analysis. Observational Study. *Wiener Klinische Wochenschrift*. Dec 2022;134(23-24):883-891. doi:https://dx.doi.org/10.1007/s00508-022-02098-9
71. Koh LP, Chua SL, Vasoo S, et al. Real-world effectiveness of sotrovimab and remdesivir for early treatment of high-risk hospitalized COVID-19 patients: A propensity score adjusted retrospective cohort study. Research Support, Non-U.S. Gov't. *Journal of Medical Virology*. 02 2023;95(2):e28460. doi:https://dx.doi.org/10.1002/jmv.28460
72. Kubiliute I, Vitkauskaite M, Urboniene J, Svetikas L, Zablockiene B, Jancoriene L. Clinical characteristics and predictors for inhospital mortality in adult COVID-19 patients: A retrospective single center cohort study in Vilnius, Lithuania. *PLoS ONE*. August 2023;18(8 August) (no pagination)e0290656. doi:https://dx.doi.org/10.1371/journal.pone.0290656
73. Lakhanpal M, Sarkar D, Kumar R, Yadav I. Reduction in the Rate of Mortality of Moderate to Severe COVID 19 Infected Patients with the use of Remdesivir - A Tertiary Care Hospital-Based Retrospective Observational Study. *Anesthesia: Essays and Researches*. Jul-Sep 2022;16(3):296-300. doi:https://dx.doi.org/10.4103/aer.aer_55_22
74. Lapadula G, Bernasconi DP, Bellani G, et al. Remdesivir Use in Patients Requiring Mechanical Ventilation due to COVID-19. *Open Forum Infectious Diseases*. 2020;7(11)doi:https://dx.doi.org/10.1093/ofid/ofaa481
75. Leding C, Bodilsen J, Brieghel C, et al. Treatment effect modifiers in hospitalised patients with COVID-19 receiving remdesivir and dexamethasone. *Infectious Diseases*. 05 2023;55(5):351-360. doi:https://dx.doi.org/10.1080/23744235.2023.2187081
76. Leegwater E, Dol L, Benard MR, et al. Rapid Response to Remdesivir in Hospitalised COVID-19 Patients: A Propensity Score Weighted Multicentre Cohort Study. *Infect Dis Ther*. Oct 2023;12(10):2471-2484. doi:10.1007/s40121-023-00874-2
77. Lim S, Schreiner P, Lifson A, Bye E, Como-Sabetti K, Lynfield R. Lower Risk of ICU Admission with Remdesivir in Patients Hospitalized with COVID-19 Pneumonia. Conference Abstract. *Open Forum Infectious Diseases*. November 2021;8(SUPPL 1):S364. doi:https://dx.doi.org/10.1093/ofid/ofab466.726
78. Lim H, Palaiodimos L, Berto CG, et al. Remdesivir in the Treatment of COVID-19: A Propensity Score-Matched Analysis from a Public Hospital in New York City Assessing Renal and Hepatic Safety. *J Clin Med*. May 31 2022;11(11)doi:10.3390/jcm11113132
79. Lucijanić M, Bušić N, Bistrović P, et al. Real-life experience with remdesivir for treatment of hospitalized coronavirus disease 2019 patients: matched case-control study from a large tertiary hospital registry. *Croat Med J*. Dec 31 2022;63(6):536-543. doi:10.3325/cmj.2022.63.536

80. Malik MI, Zafar SAF, Malik M, et al. The utility of remdesivir in SARS-CoV-2: A single tertiary care center experience from a developing country. *Explor Res Clin Soc Pharm*. Mar 2022;5:100107. doi:10.1016/j.rcsop.2022.100107
81. Marocco R, Del Borgo C, Tortellini E, et al. Use of Remdesivir in Patients with SARS-CoV-2 Pneumonia in a Real-Life Setting during the Second and Third COVID-19 Epidemic Waves. *Viruses*. Apr 11 2023;15(4)doi:10.3390/v15040947
82. Marx K, Goncarova K, Fedders D, et al. Clinical outcomes of hospitalized COVID-19 patients treated with remdesivir: a retrospective analysis of a large tertiary care center in Germany. *Infection*. February 2023;51(1):97-108. doi:https://dx.doi.org/10.1007/s15010-022-01841-8
83. Metchurtchlishvili R, Chkhartishvili N, Abutidze A, et al. Effect of remdesivir on mortality and the need for mechanical ventilation among hospitalized patients with COVID-19: real-world data from a resource-limited country. *Int J Infect Dis*. Apr 2023;129:63-69. doi:10.1016/j.ijid.2023.01.021
84. Mohanty B, Sunder A, Satyanarayan B, Kumar M, Shukla R, Ahmed A. Success rate of Remdesivir, Convalescent Plasma, and Tocilizumab in moderate to severe Covid-19 pneumonia: our experience in a tertiary care center. *J Family Med Prim Care*. Nov 2021;10(11):4236-4241. doi:10.4103/jfmpc.jfmpc_578_21
85. Monardo R, Mastrangelo A, Galli L, et al. Real-life use of High-Dose Anakinra in Patients with COVID-19 Treated with Remdesivir. *Future Virology*. 2024/01/01 2024;19(1):11-17. doi:10.2217/fvl-2023-0132
86. Mozaffari E, Chandak A, Zhang Z, et al. Remdesivir Treatment in Patients Hospitalized with COVID-19: A Comparative Analysis of In-Hospital All-Cause Mortality. *Open Forum Infectious Diseases*. 2021;8(Supplement_1):S27-S28. doi:10.1093/ofid/ofab466.038
87. Mozaffari E, Chandak A, Liang S, et al. Early treatment with remdesivir and in-hospital mortality among hospitalised COVID-19 patients in the real-world setting. Conference Abstract. 2021;
88. Mozaffari E, Chandak A, Zhang Z, et al. Remdesivir Treatment in Hospitalized Patients With Coronavirus Disease 2019 (COVID-19): A Comparative Analysis of In-hospital All-cause Mortality in a Large Multicenter Observational Cohort. *Clinical Infectious Diseases*. 08 24 2022;75(1):e450-e458. doi:https://dx.doi.org/10.1093/cid/ciab875
89. Mozaffari E, Chandak A, Gottlieb RL, et al. Remdesivir Reduces Mortality in Hospitalized Covid-19 Patients across Variant Eras. Conference Abstract. *Topics in Antiviral Medicine*. April 2023;31(2):218-219.
90. Mozaffari E, Chandak A, Gottlieb RL, et al. Remdesivir is associated with reduced readmission after COVID-19 hospitalization. Conference Abstract. 2023;
91. Ngo DQ, Hamid K, Rana H, et al. A Retrospective Study of Dexamethasone, Remdesivir, and Baricitinib in Severe COVID-19. *The Canadian Journal of Infectious Diseases & Medical Microbiology*. 2022;2022:9209618. doi:https://dx.doi.org/10.1155/2022/9209618
92. Panda R, Singh P, Jain G, et al. Effect of Remdesivir Administration on Occurrence of Major Adverse Cardiac Events in Critically Ill COVID-19 Pneumonia: A Retrospective Observational Study. *Indian J Crit Care Med*. Sep 2022;26(9):993-999. doi:10.5005/jp-journals-10071-24189
93. Pantazopoulos I, Mavrovounis G, Dimeas G, et al. Remdesivir-induced Bradycardia is not Associated with Worse Outcome in Patients with COVID-19: A Retrospective Analysis. *Am J Cardiovasc Drugs*. Nov 2022;22(6):705-710. doi:10.1007/s40256-022-00547-4
94. Paranjape N, Husain M, Priestley J, Koonjah Y, Watts C, Havlik J. Early Use of Remdesivir in Patients Hospitalized With COVID-19 Improves Clinical Outcomes: A Retrospective Observational Study. *Infect Dis Clin Pract (Baltim Md)*. Sep 2021;29(5):e282-e286. doi:10.1097/ipc.0000000000001023
95. Pham HT, Mai-Phan TA, Vu AK, Truong TH, Tran MH. Clinical use of remdesivir in COVID-19 treatment: a retrospective cohort study. *BMJ Open*. Jun 9 2023;13(6):e070489. doi:10.1136/bmjopen-2022-070489

96. Pivato L, Bettio M, Mengato D, et al. 6ER-025 Real-life use of remdesivir in hospitalised COVID-19 patients with severe pneumonia: an observational study from an Italian university hospital. *European Journal of Hospital Pharmacy*. 2022;29(Suppl 1):A176-A176. doi:10.1136/ejhpharm-2022-eahp.370
97. Platzer M, Totschnig D, Karolyi M, Clodi-Seitz T, Wenisch C, Zoufaly A. The effect of early remdesivir administration in COVID-19 disease progression in hospitalised patients. *Wien Klin Wochenschr*. Jun 17 2024;doi:10.1007/s00508-024-02377-7
98. Poliseno M, Gallo C, Cibelli DC, et al. Efficacy and Safety of Remdesivir over Two Waves of the SARS-CoV-2 Pandemic. *Antibiotics (Basel)*. Dec 1 2021;10(12)doi:10.3390/antibiotics10121477
99. Rai DK. Effectiveness of remdesivir on hospital stay duration and mortality in patients with COVID-19 infection: A single-center retrospective observational study. *Lung India*. Jan-Feb 2022;39(1):85-88. doi:10.4103/lungindia.lungindia_268_21
100. Rawat M, Choudhary S, Gaur A, et al. Comparison of Remdesivir As Monotherapy or in Combination with Steroids in Treating COVID-19 Patients. *Value Health*. Jul 2022;25(7):S581-2. doi:10.1016/j.jval.2022.04.1555
101. Razzaq MA, Bhutta SH, Alam M, Nizam K, Siddique MU, Sharif MI. Comparison of Clinical Improvement with VS without Remdesivir Treatment in Hospitalized Patients with COVID-19. 2022:
102. Read C, Claire R, Elvidge J, et al. Decision Making and the Questions Raised When Real World Evidence From a Federated Data Network Conflicts With RCT Data: A Use Case on COVID-19 Treatments. *Value in Health*. 2023;26(12):S508.
103. Rivera CG, Chesdachai S, Draper EW, et al. Clinical outcomes of COVID-19 treated with remdesivir across the continuum of care. *Influenza Other Respir Viruses*. May 2023;17(5):e13136. doi:10.1111/irv.13136
104. Russo A, Binetti E, Borrazzo C, et al. Efficacy of Remdesivir-Containing Therapy in Hospitalized COVID-19 Patients: A Prospective Clinical Experience. *J Clin Med*. Aug 24 2021;10(17)doi:10.3390/jcm10173784
105. Russo P, Tacconelli E, Olimpieri PP, et al. Mortality in SARS-CoV-2 Hospitalized Patients Treated with Remdesivir: A Nationwide, Registry-Based Study in Italy. *Viruses*. May 31 2022;14(6)doi:10.3390/v14061197
106. Said MZ-M, Ramirez C, Almaguer A, et al. 443: REMDESIVIR USE IN HOSPITALIZED COVID-19 PATIENTS. *Critical Care Medicine*. 2023;51(1):208. doi:10.1097/01.ccm.0000907500.59357.57
107. Sattoju N, Gattu S, Merugu SS, Anneboina V, Ganapaka SR. Dexamethasone, dexamethasone + remdesivir in treating moderate to severe COVID-19: retrospective observational cohort study. *J Infect Dev Ctries*. Jul 27 2023;17(7):953-960. doi:10.3855/jidc.17971
108. Sellers J, Chang J, Jones J, Hintze TD. Patients with moderate to severe COVID-19 outcomes on remdesivir according to baseline 4C mortality score. *Pulm Pharmacol Ther*. Feb 2023;78:102188. doi:10.1016/j.pupt.2022.102188
109. Senthiappan RP, Dambal AA, Mohan K, Aithal KR, Parakh RK, Vuppumalla B. Clinical Outcomes of Remdesivir in Moderate and Severe Cases of COVID-19: A Retrospective Cohort Study. *Journal of Clinical & Diagnostic Research*. 2023;17(1)
110. So JM, Umeh C, Noriega S, Stratton E, Aseri M, Gupta RC. Use of Baricitinib in Combination With Remdesivir and Steroid in COVID-19 Treatment: A Multicenter Retrospective Study. *Cureus*. Dec 2021;13(12):e20620. doi:10.7759/cureus.20620
111. Spagnuolo V, Voarino M, Tonelli M, et al. Impact of Remdesivir on SARS-CoV-2 Clearance in a Real-Life Setting: A Matched-Cohort Study. *Drug Des Devel Ther*. 2022;16:3645-3654. doi:10.2147/dddt.S369473
112. Ughi N, Bernasconi DP, Del Gaudio F, et al. Effectiveness and Safety of Remdesivir in Treating Hospitalised Patients with COVID-19: A Propensity Score Analysis of Real-Life Data from a Monocentric

Observational Study in Times of Health Emergency. *Clin Drug Investig*. Oct 2023;43(10):763-771. doi:10.1007/s40261-023-01304-4
113. Umeh CA, Maguwudze S, Kaur H, et al. Bradycardia and Outcomes in COVID-19 Patients on Remdesivir: A Multicenter Retrospective Study. *Cardiology Research*. Jun 2023;14(3):192-200. doi:https://dx.doi.org/10.14740/cr1493
114. Viray DD, Cajulao TP, Demot B, Bartolo S, Feliciano D. CLINICAL EFFECTIVENESS AND ADVERSE EVENTS OF ADJUNCTIVE REMDESIVIR ADMINISTRATION VS STANDARD OF CARE IN ADULTS WITH SEVERE AND CRITICAL COVID-19. *Int J Infect Dis*. May 2023;130:S143. doi:10.1016/j.ijid.2023.04.352
115. Williams KN, Gauthier TP, Baker SW, Cua J. Real World Evaluation of Clinical Outcomes for Short-Duration Remdesivir Therapy in Hospitalized Patients with Mild-Moderate COVID-19. *Open Forum Infect Dis*. Dec 2023;10(Suppl 2)doi:10.1093/ofid/ofad500.518
116. Wong CKH, Lau KTK, Au ICH, et al. Optimal Timing of Remdesivir Initiation in Hospitalized Patients with Coronavirus Disease 2019 (COVID-19) Administered with Dexamethasone. *Clinical Infectious Diseases*. 01 Jul 2022;75(1):E499-E508. doi:https://dx.doi.org/10.1093/cid/ciab728
117. Wong CKH, Lau KTK, Au ICH, Xiong X, Lau EHY, Cowling BJ. Clinical Improvement, Outcomes, Antiviral Activity, and Costs Associated With Early Treatment With Remdesivir for Patients With Coronavirus Disease 2019 (COVID-19). *Clin Infect Dis*. Apr 28 2022;74(8):1450-1458. doi:10.1093/cid/ciab631
118. Wu B, Luo M, Wu F, He Z, Li Y, Xu T. Acute Kidney Injury Associated With Remdesivir: A Comprehensive Pharmacovigilance Analysis of COVID-19 Reports in FAERS. *Front Pharmacol*. 2022;13:692828. doi:10.3389/fphar.2022.692828
119. Olender SA, Walunas TL, Martinez E, et al. Remdesivir Versus Standard-of-Care for Severe Coronavirus Disease 2019 Infection: An Analysis of 28-Day Mortality. *Open Forum Infectious Diseases*. Jul 2021;8(7):ofab278. doi:https://dx.doi.org/10.1093/ofid/ofab278
120. Paules CI, Gallagher SK, Rapaka RR, et al. Remdesivir for the Prevention of Invasive Mechanical Ventilation or Death in Coronavirus Disease 2019 (COVID-19): a Post Hoc Analysis of the Adaptive COVID-19 Treatment Trial-1 Cohort Data. Journal article. *Clinical infectious diseases*. 2022;74(7):1260 - 1264. doi:10.1093/cid/ciab695
121. Henao-Restrepo AM, Pan H, Peto R, et al. Remdesivir and three other drugs for hospitalised patients with COVID-19: final results of the WHO Solidarity randomised trial and updated meta-analyses. *The Lancet*. 21 May 2022;399(10339):1941-1953. doi:https://dx.doi.org/10.1016/S0140-6736%2822%2900519-0
122. Fintzi J, Bonnett T, Sweeney DA, et al. Deconstructing the Treatment Effect of Remdesivir in the Adaptive Coronavirus Disease 2019 (COVID-19) Treatment Trial-1: Implications for Critical Care Resource Utilization. *Clinical Infectious Diseases*. 07 06 2022;74(12):2209-2217. doi:https://dx.doi.org/10.1093/cid/ciab712
